# MYB31/MYB42 Syntelogs Exhibit Divergent Regulation of Phenylpropanoid Genes in Maize, Sorghum and Rice

**DOI:** 10.1038/srep28502

**Published:** 2016-06-22

**Authors:** Tina Agarwal, Erich Grotewold, Andrea I. Doseff, John Gray

**Affiliations:** 1 Department of Biological Sciences, University of Toledo, Toledo, OH 43606 USA; 2 Center for Applied Plant Sciences (CAPS) and Department of Molecular Genetics, The Ohio State University, 1060 Carmack Rd, 012 Rightmire Hall, Columbus, OH 43210, USA; 3 Department of Molecular Genetics, The Ohio State University, Columbus, Ohio, 43210 USA; 4 Department of Physiology and Cell Biology and The Heart and Lung Research Institute, The Ohio State University, Columbus, Ohio, 43210 USA

## Abstract

ZmMYB31 and ZmMYB42 are R2R3-MYB transcription factors implicated in the regulation of phenylpropanoid genes in maize. Here, we tested the hypothesis that the regulatory function of MYB31 and MYB42 is conserved in other monocots, specifically in sorghum and rice. We demonstrate that syntelogs of MYB31 and MYB42 do bind to phenylpropanoid genes that function in all stages of the pathway and in different tissues along the developmental gradient of seedling leaves. We found that *caffeic acid O-methyltransferase* (*COMT1*) is a common target of MYB31 and MYB42 in the mature leaf tissues of maize, sorghum and rice, as evidenced by Chromatin immunoprecipitation (ChIP) experiments. In contrast, *4-coumarate-CoA ligase* (*4CL2*), *ferulate-5-hydroxylase* (*F5H*), and *caffeoyl shikimate esterase* (*CSE*), were targeted by MYB31 or MYB42, but in a more species-specific fashion. Our results revealed MYB31 and MYB42 participation in auto- and cross-regulation in all three species. Apart from a limited conservation of regulatory modules, MYB31 and MYB42 syntelogs appear to have undergone subfunctionalization following gene duplication and divergence of maize, sorghum, and rice. Elucidating the different regulatory roles of these syntelogs in the context of positive transcriptional activators may help guide attempts to alter the flux of intermediates towards lignin production in biofuel grasses.

The desire to increase the suitability of plant residues (stover) as a biofuel source has led to a renewed interest in understanding and manipulating the regulation of the general phenylpropanoid pathway from which lignin is produced^1^,^2^,^3^,^4^. Several transcription factors (TFs) that regulate lignin biosynthesis, including transcriptional activators and repressors, have been identified of which several are MYB TFs^1^,^5^,^6^. In particular ZmMYB31 and ZmMYB42, which belong to the R2R3-MYB subgroup 4, harbor a pdLNL[D/E]Lxi[G/S] (EAR) motif associated with transcription repression^7^, and are thought to act as repressors of lignin biosynthesis^8^,^9^,^10^. In maize the R2R3-MYB regulator C1 cooperates with the basic helix–loop–helix (bHLH) factor R to activate the expression of phenylpropanoid biosynthetic genes in a coordinate fashion^11^. ZmMYB31 represses *COMT1* expression in transient expression assays, occupies the promoter of *F5H in vivo*, and could counter the positive regulation of the maize C1/R regulatory complex^8^. Recently, ChIP-PCR evidence suggested enriched binding of ZmMYB31 and ZmMYB42 to other phenylpropanoid promoters using entire maize leaves as source material^12^. ZmMYB11, a ZmMYB31 paralog, interacts directly with the TIFY type co-regulator ZmZML2 on the maize *COMT1* promoter. Furthermore the jasmonate-signaling cascade triggers the degradation of the ZmMYB11/ZmZML2 complex in response to wounding, thus leading to *COMT1* de-repression. Bifluorescence complementation assays (BiFC) provided evidence that ZmZML2 interacts with ZmMYB31 and ZmMYB42 in maize protoplasts^12^.

Such studies suggest that MYB11/31/42 functions are dynamically regulated *in vivo*. How lignin biosynthesis genes are coordinately regulated by MYB11/31/42 is not well understood and tissue specific events are often overlooked as entire leaves are used for analysis. The presence of several members of target gene families increases the complexity of gene regulation. For example, there are seven *CCR*, seven *CAD*, and eight *4CL* genes in the maize phenylpropanoid pathway, allowing for the possibility of redundant or partially redundant functions, as suggested in RNAi knock-down of *coumarate 3-hydroxylase-1* (*C3H*) in maize^13^. Moreover, the recent identification of caffeoyl shikimate esterase (CSE) leading to the hydrolysis of caffeoyl shikimate into caffeate and forming S + G monolignols, supports the existence of additional regulatory mechanisms^14^. Whether *CSE* is involved in the phenylpropanoid pathway of monocots, however, is not yet defined, illustrating our limited understanding of the lignin biosynthetic pathway.

Whereas MYB11/31/42 have been implicated in the negative regulation of phenylpropanoid genes in maize, until recently, positive regulators of these genes were only studied in dicots^4^,^15^,^16^. Recent evidence suggests that two R2R3 MYB TFs namely ZmMYB152 (ZmMYB111) (GRMZM2G104551) and ZmMYB5 (ZmMYB148) (GRMZM2G097636) may play a role in the positive regulation of phenylpropanoid genes but their function has not been confirmed *in planta*^17^. In addition, ecotopic expression of sorghum *SbMYB60* (Sb04g031110) was associated with higher expression levels of genes involved in monolignol biosynthesis, and led to changes in lignin composition^18^. Thus the regulatory network governing phenylpropanoid genes in monocots appears to involve a dynamic balance of positive and negative MYB TF regulators and other regulators are likely to be discovered.

Comparative approaches that examine the conservation or divergence of regulatory modules across species can help reconstruct regulatory networks as well as provide insights into the evolution of gene regulatory processes and their contribution to species adaptation^19^,^20^. In this study, we used available genome sequences of maize, sorghum and rice, to identify *ZmMYB31* and *ZmMYB42* gene orthologs, study their expression patterns and test if there is conservation or divergence of their target genes along the developmental gradient of seedling leaves^21^. Using ChIP-PCR, we identified that there are at least four genes (*COMT1*, *F5H*, *4CL2*, and *CSE*) regulated by MYB31 and MYB42 in these three species. The patterns of TF binding were both tissue- and species- specific with little obvious conservation of regulatory modules. These TFs exhibited repressor activity as evidenced when transiently expressed in maize protoplasts. Both MYB31 and MYB42 were found to participate in cross-regulatory and auto-regulatory circuits. MYB42 regulates both *MYB31* and *MYB42* genes in all three species, albeit in different leaf tissues. In rice, MYB31 exhibits weak binding to both *OsMYB31* and *OsMYB42*, an activity apparently absent in maize and sorghum. Taken together, we have uncovered novel conserved and specialized regulatory activities of MYB31 and MYB42 across three grasses.

## Results

### Identification of *ZmMYB31* and *ZmMYB42* syntelogs in rice and sorghum

**Figure 1:**
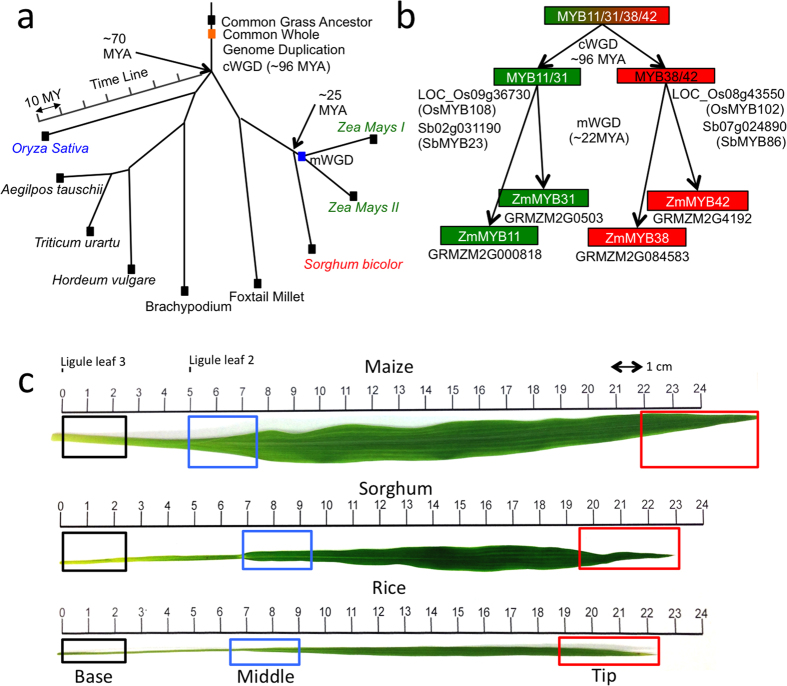
Identification of ZmMYB31 and ZmMYB42 syntelogs in sorghum and rice and the generation of specific antisera. (**a**) Schematic representing the evolution of grass species from a common ancestor (adapted with permission from^22^). Arrows indicate the estimated time since rice, sorghum and maize have diverged from a common ancestor. (**b**) Schematic representing the inferred relationship between *ZmMYB31* and *ZmMYB42* and homologous genes in maize, sorghum and rice as a result of an ancient common whole genome duplication and recent maize specific whole genome duplication. (**c**) Scaled image of leaf samples used for comparative gene expression (qRT-PCR) and TF binding (ChIP-PCR) measurements in this study. Black, blue, and red boxes indicate the 2.5 cm sections that were defined as leaf base, mid-leaf, and leaf tip sections respectively.

To determine the regulatory roles of MYB31 and MYB42 in sorghum and rice, which diverged from maize about 25 and 70 million years ago (MYA) respectively (Fig. 1a)^22^, the corresponding genes in these grasses were first identified through homology and synteny. Grass genomes share a largely common set of homologous genes as a result of a common whole-genome duplication (cWGD) event that occurred prior to their divergence about 96 MYA (Fig. 1a)^22^,^23^. We can more precisely describe such homologs as ohnologs when they result from a WGD event and as syntelogs if there is evidence that they are derived from the same ancestral genomic region. Thus, there are ohnologs of *ZmMYB31* and *ZmMYB42* in both rice and sorghum that arose as a result of the cWGD (Fig. 1b). In the maize lineage, another whole genome duplication occurred (mWGD)^22^, giving rise to two more ohnologs of *ZmMYB31* and *ZmMYB42*, which are *ZmMYB11* and *ZmMYB38* respectively (Fig. 1a).

The gene encoding *ZmMYB31* (GRMZM2G050305) on chromosome 2 was found to exist within a large syntenic block with the sorghum Sb02g031190 (*SbMYB23*) gene on chromosome 2 and the rice LOC_Os09g36730 (*OsMYB108*) gene on chromosome 9 (Supplementary Fig. S1a). *ZmMYB11* (GRMZM2G000818) and *ZmMYB31* originated as a result of a mWGD, and are therefore considered ohnologs. *ZmMYB11* exhibits a lower synteny block score with sorghum Sb02g031190 and rice LOC_Os09g36730 (see Supplementary Fig. S1b). *ZmMYB42* (GRMZM2G419239) on chromosome 4 exists within a smaller syntenic region with sorghum Sb07g024890 (*SbMYB86*) on chromosome 7 and rice LOC_Os08g43550 (*OsMYB102*) gene on chromosome 8 (see Supplementary Fig. S1c). Sorghum and rice syntelogs will be referred hereafter as *SbMYB31* or *OsMYB31* and *SbMYB42* or *OsMYB42*, respectively (Fig. 1b). *ZmMYB38* (GRMZM2G0845), an ohnolog of *ZmMYB42*, exhibits a higher synteny block score with the sorghum and rice homologous genes (see Supplementary Fig. S1d). *ZmMYB42* was chosen for this study because of previous work linking this R2R3-MYB protein to the regulation of the phenylpropanoid pathway^8^,^9^,^10^. Although both *ZmMYB31* and *ZmMYB42* have ohnologs in maize (*ZmMYB11* and *ZmMYB38*, respectively), the mWGD arose after maize diverged from sorghum and rice so these latter species do not exhibit these gene duplications (Fig. 1b).

We also identified syntelogs of R2R3 MYB TFs that have been recently implicated in the positive regulation of phenylpropanoid genes in maize and sorghum^17^,^18^. The *ZmMYB5* and *ZmMYB13* genes both appear to be ohnologs of the unique *SbMYB76* and *OsMYB60* genes (see Supplementary Fig. S2a,b and Supplementary Table S4). The *ZmMYB19* and *ZmMYB152* genes do not appear to have duplicates in maize but exhibit strong synteny with *SbMYB60/OsMYB33* and *SbMYB22/OsMYB107* respectively (Supplementary Fig. S2c,d).

To determine whether *MYB31* and *MYB42* syntelogs have conserved regulatory roles in the phenylpropanoid pathway, we generated antisera that detect MYB31, MYB42 proteins. For this purpose, we identified the region of least conservation by multiple sequence alignment (see Supplementary Fig. S3a). The region of ZmMYB31 chosen as antigen appears to have undergone a large deletion in ZmMYB11, which adds to the specificity of the antibodies. The anti-ZmMYB31 antiserum did not exhibit cross reactivity with ZmMYB11 overexpressed in *E. coli* (see Supplementary Fig. S3c). The chosen unique region of each *MYB31* and *MYB42* gene from maize, sorghum and rice was cloned into a bacterial expression vector with a 6XHis tag and fusion proteins were produced in *E. coli*, affinity purified, and used to generate polyclonal antisera in rabbits. The antisera were then tested for specificity against the corresponding regions of the proteins chosen for antisera generation (see Supplementary Fig. S3a) from the various grasses. We found that anti-SbMYB31 and anti-OsMYB31 lack cross reactivity with SbMYB42 and OsMYB42 (see Supplementary Fig. S3b). However the anti-MYB42 antisera exhibited weak cross reactivity with the MYB31 proteins in all three species. Therefore, the anti-MYB42 antisera were further affinity purified, and further studies revealed no cross reactivity with MYB31 (see Supplementary Fig. S3b), or with ZmMYB38 (see Supplementary Fig. S3c) that were overexpressed in *E. coli*.

### Co-expression of maize and rice biosynthetic and regulatory genes along the leaf developmental gradient

**Figure 2:**
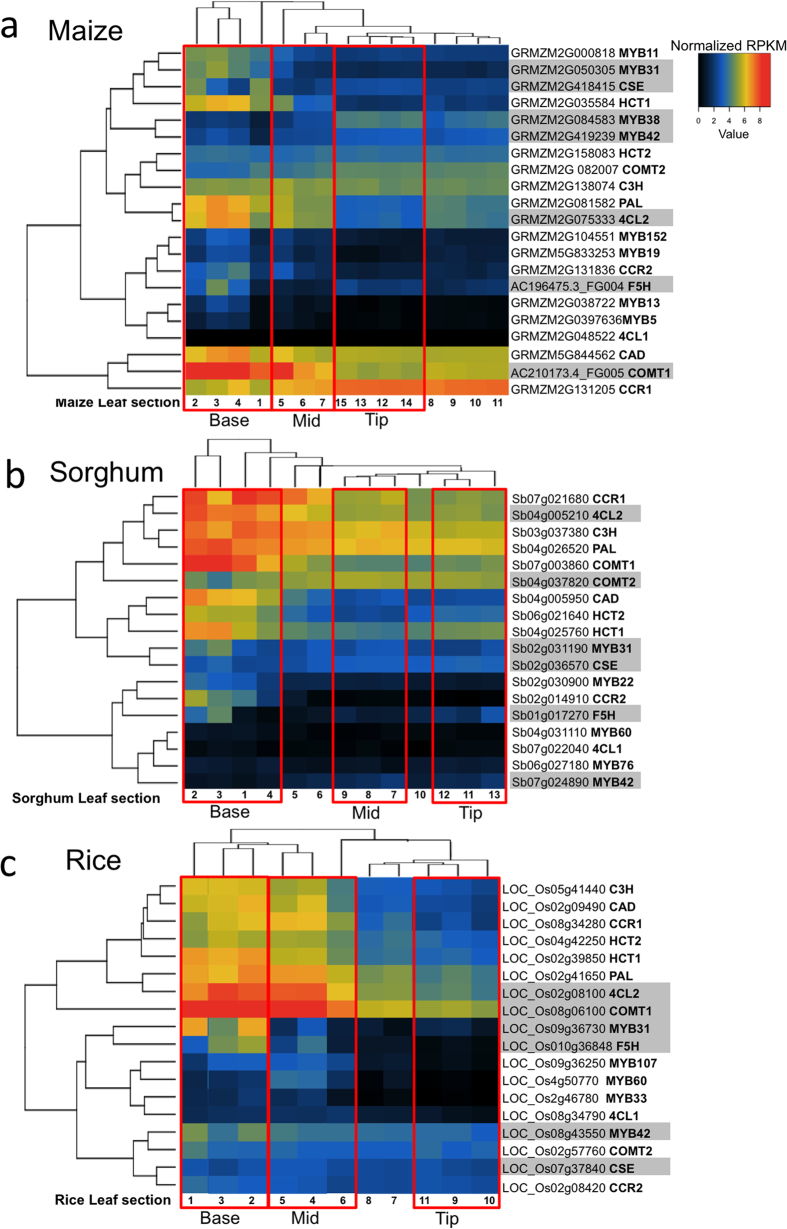
*In silico* expression analysis of phenylpropanoid pathway genes and regulatory MYB genes in maize, sorghum, and rice leaves. (**a–c**) Heat map representation of tissue-specific expression levels of phenypropanoid genes and MYB regulatory genes (*ZmMYB11*, *ZmMYB31*, *ZmMYB38*, *ZmMYB42, ZmMYB5, ZmMYB13, ZmMYB19*, and *ZmMYB152, SbMYB31, SbMB42, SbMYB60, SbMYB76* and *SbMYB22, or OsMYB31, OsMYB42, OsMYB33, OsMYB60 and OsMYB107*) in maize (**a**), sorghum (**b**)and rice (**c**) leaves respectively. The genes highlighted in gray are those genes selected for promoter occupancy studies. Expression levels are represented as colors ranging from Red (highest expression) to black (undetectable expression). Fifteen, thirteen, or eleven columns correspond to RNA-Seq libraries. The red boxes indicate the tissues considered as base, middle, and tip tissues for further study. Each row represents a gene considered in this study. Hierarchical clustering of leaf sections with similar expression patterns represented by the dendrogram above the heat map. Hierarchical clustering of genes with similar expression patterns presented by the dendrogram at the left of the heat map.

To define the regulatory landscape that exists for the phenylpropanoid pathway in leaves, we analyzed the expression and co-expression patterns of 15 phenylpropanoid genes and *MYB31/42* using existing RNA-Seq datasets from previous studies in maize, sorghum, and rice^24^,^25^. First, the pattern of gene expression in 15 maize leaf segments, including leaf base, middle and tip was analyzed using hierarchal clustering (Fig. 2a, top dendrogram). Four basal leaf segments clustered together and collectively exhibited a higher level of gene expression than in more mature tissues (Fig. 2a, boxed regions and Supplemental Fig. S4). A majority of phenylpropanoid genes exhibited their lowest level of expression in the tip of the leaf (Fig. 2a and Supplemental Fig. S4). This was true for the four genes (*COMT1*, *4CL2*, *CSE* and *F5H*) later chosen for promoter occupancy studies. Of these, *ZmCOMT1* and *Zm4CL2* exhibited amongst the highest overall levels of expression whereas *ZmF5H* and *ZmCSE* exhibited moderate expression in the leaf base and very low expression in the leaf tip (Fig. 2a and Supp. Fig. S4). Co-expression analysis in sorghum and rice revealed a cluster of seven genes *COMT1, 4CL2, CCR1*, *CAD*, *HCT1*, *PAL*, and *F5H* with lowering levels of expression in the more mature mid leaf and leaf tip regions beyond the ligule (Fig. 2b,c and Supp. Fig. S4). There were only a few exceptions to this trend namely the *SbCSE and OsCSE* genes, which exhibited a low and very uniform expression pattern along the leaf gradient (Fig. 2b,c) and the *4Cl1* gene which exhibited little or no expression in leaves of all three species (Fig. 2a–c).

**Figure 3:**
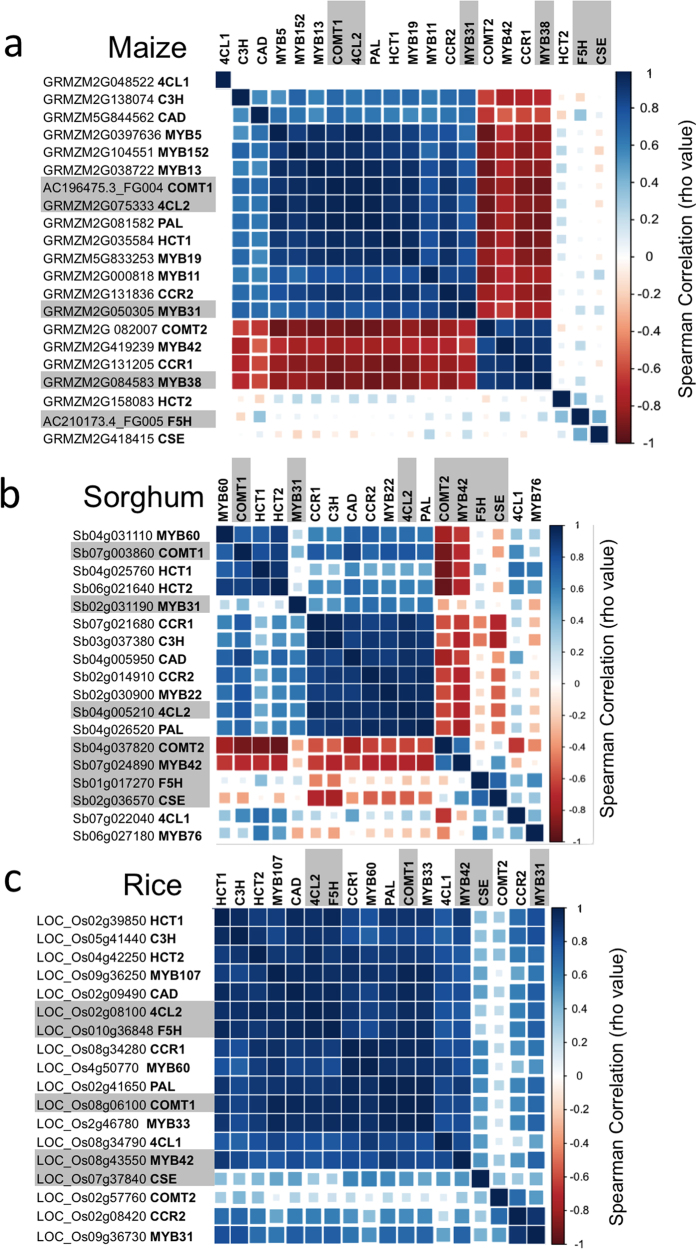
*In silico* co-expression analysis of phenylpropanoid pathway genes and regulatory MYB genes in maize, sorghum, and rice leaves. (**a–c**) Heat map representation of the Spearmen’s correlation coefficient of expression levels (log2 RPKM values) for phenylpropanoid and select *MYB* regulatory genes in 15, 13, or 11 leaf sections in maize (**a**), sorghum (**b**), and rice (**c**) respectively. The scale represents signal intensity of the normalized expression data. Dark blue color indicates high correlation between gene expression patterns, while white cell indicates absence of correlation, and dark red color indicates negative correlation. Actual ρ and *p* values of the Spearman correlation are provided in Supplementary Tables S5 and S6 for maize and rice, respectively.

The expression profile of the *MYB31/42* regulatory factors was also analyzed. In maize, the duplicate genes *ZmMYB11* and *ZmMYB31* were co-expressed (ρ = 0.83, p < 0.01) with highest expression occurring in the leaf base. This contrasted with *ZmMYB38* and *ZmMYB42*, which were also co-expressed (ρ = 0.9, p < 0.01), but exhibited highest expression in the leaf tip. The expression of the *ZmMYB11/31* gene pair positively correlated with the *ZmPAL*, *ZmF5H*, *ZmCAD*, *Zm4Cl2, ZmHCT1*, and *ZmCCR2* clade (Fig. 3a and Supplementary Table S4), whose mRNA accumulate at higher levels in the immature leaf base than in the mature leaf tip (Fig. 2a). In contrast, expression of the *ZmMYB38/42* pair negatively correlated with most phenylpropanoid genes (Fig. 3a). This inverse correlation is consistent with a proposed negative regulatory role for *ZmMYB38/42* in leaf tissues. In sorghum which lacks the more recent MYB duplication event seen in maize, the *SbMYB31* and *SbMYB42* genes are expressed at low levels in the leaf as is expected for transcription factors. *SbMYB42* expression is negatively correlated with ten of the phenylpropanoid genes (ρ ≤ −0.6, p ≤ 0.02). In rice, which also lacks the more recent MYB duplication event seen in maize, the highest level of expression of *OsMYB42* is in the leaf base (Fig. 2c), which positively correlated with the expression pattern of a majority of phenylpropanoid structural genes (Fig. 3c). *OsMYB31* exhibited very low levels of expression in leaf tips and thus may be playing a reduced regulatory role compared to *OsMYB42* in mature leaf tissues (Fig. 2c).

We also examined the co-expression of phenylpropanoid genes with MYB TFs recently proposed to act as positive regulators. The expression of the *ZmMYB19*, *SbMYB60*, and *OsMYB33* syntelogs exhibited a positive correlation (ρ ≥ 0.6, p ≤ 0.02), with that of *4CL2*, *COMT1*, *CCR2*, *HCT1*, and *C3H* targets in all three species (Fig. 3a–c and Supp. Tables S5–7). Similarly, the *ZmMYB13/5* and *OsMYB60* syntelogs were strongly correlated with the same set of genes however expression of the sorghum syntelog (*SbMYB76*) which was expressed at very low levels along the leaf gradient (Fig. 2b) did not positively correlate with any phenylpropanoid targets (Fig. 3a–c and Supp. Tables S5–7). The *ZmMYB152*, *SbMYB22*, and *OsMYB107* syntelogs were strongly positively correlated with an overlapping set of targets namely *4CL2*, *COMT1*, *CCR2*, *CAD*, *HCT1*, and *PAL* (ρ ≥ 0.8, p ≤ 0.01) in all three species. In maize and sorghum but not rice, expression of the proposed positive regulators, (except for *SbMYB76*) was negatively correlated with the expression of proposed negative regulators i.e. MYB42/38 homologs (ρ ≤ 0.7, p ≤ 0.01) (Fig. 3a,b and Supp. Tables S5–6). In general the proposed positive MYB regulators were co-expressed with a subset of target phenylpropanoid genes and the repertoire of targets differed slightly across species.

In summary, analysis of the RNA-seq data from rice provided evidence of co-expression of most of the phenylpropanoid genes with a majority exhibiting lowest expression in the mature leaf tip tissue. The expression of a majority of phenylpropanoid genes was positively and negatively correlated with *ZmMYB31* and *ZmMYB42* expression respectively in maize. In sorghum the expression of most phenylpropanoid genes also negatively correlated with *SbMYB42* expression but only a weak positive correlation with SbMYB31 was observed. In rice, the expression of both OsMYB42 and OsMYB31 was positively correlated with the expression of phenylpropanoid genes.

### Differential accumulation of MYB31 and MYB42 and target phenylpropanoid mRNAs in maize, sorghum, and rice seedling leaves

**Figure 4:**
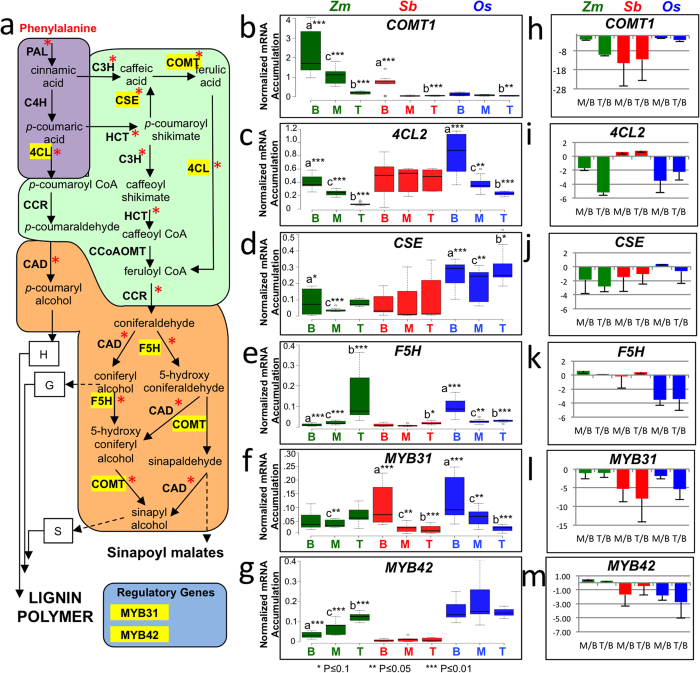
Survey of MYB31 and MYB42 gene expression in seedling leaves from maize, sorghum and rice. (**a**) Schematic of phenylpropanoid pathway illustrating the functional steps of the genes whose expression was determined along the leaf gradient (in yellow). Common early branch (purple shading): PAL, phenylalanine ammonia-lyase; C4H, cinnamate 4-hydroxylase; 4CL, 4-coumarate-CoA ligase. The monolignol biosynthetic (ester to aldehyde) middle branch (green shading): C3H, 4-coumarate 3-hydroxylase; COMT, caffeic acid *O*-methyltransferase; CSE, caffeoyl shikimate esterase; HCT, hydroxycinnamoyl-CoA shikimate/quinate hydroxycinnamoyl transferase; CCoAOMT, caffeoyl-CoA o-methyltransferase; and CCR, cinnamoyl-CoA reductase. The monolignol biosynthetic (aldehyde to alcohol) “late” branch (orange shading): F5H, ferulate-5-hydroxylase; CAD, cinnamyl alcohol dehydrogenase. Regulatory genes MYB31 and MYB42 are shown separate from the biochemical pathway (blue shading). (**b–g**) mRNA accumulation levels of *COMT1* (**b**)*, CSE1* (**c**)*, 4-CL2* (**d**), *F5H* (**e**), *MYB31* (**f**) AND *MYB42* (**g**) in the leaf base (B) midleaf (M) or leaf tip (T) regions of maize, sorghum, and rice leaves. mRNA accumulation was determined by qRT-PCR and normalized to a reference gene (tubulin in maize, ubiquitin in sorghum and rice). Expression levels (2^−ΔCT^ values)^54^ were plotted as boxplots and each boxplot represents three biological replicates with three technical replicates each. Statistically significant changes in mRNA accumulation along the leaf gradient are shown as a: leaf base versus mid leaf, b: leaf base versus leaf tip, and c: mid leaf versus leaf tip. Asterisks represent statistical significance of the observed differences between tissues as *P ≤ 0.1,**P ≤ 0.05, and ***P ≤ 0.01, (2 tailed *t*-test). (**h–m**) Relative levels of *Comt1, 4-CL2*, *Cse1, F5H*, *MYB31* and *MYB42* gene expression in leaf middle versus leaf base (M/B) or leaf tip versus leaf base (T/B) in maize, sorghum, and rice. Gene expression values as reported in b-g are expressed as a ratio to expression in the basal tissue of the leaf. Each graph represents the average of three biological replicates. Error bars represent the standard deviation of the three biological replicates.

Next, we profiled the steady state mRNA levels of phenylpropanoid genes in the base, mid leaf, and leaf tip tissues of sorghum seedling leaves for comparison with maize and rice (Fig. 1c, black, blue and red boxed regions). The same material was subsequently used in MYB31/42 promoter occupancy studies. The expression of nine orthologous genes, corresponding to enzymatic steps in the maize phenylpropanoid pathway, were studied (Fig. 4a, indicated with an asterisk and see Supplementary Table S4). Of the nine genes studied, *COMT1*, *4CL2*, *F5H*, and *CSE* exhibited consistent changes in relative mRNA accumulation along the leaf axis in one or more of the species examined (Fig. 4b–e). These four genes were chosen for further study and also are representative of genes that function at different stages (early to middle [*4CL2*], middle [*CSE*], and middle to late [*COMT1, F5H*]) in the pathway (Fig. 4a).

With a few exceptions it was found using qRT-PCR that the phenylpropanoid and regulatory genes followed a general expression pattern aligned to the transcriptomic studies. In general the lowest levels of phenylpropanoid target gene mRNA accumulation were observed in the mature leaf tip region. In sorghum and maize, the lowest steady state mRNA levels of *COMT1* occurred in the leaf tip but a more uniform expression was seen along rice leaves (Fig. 4b,h). *4CL2* mRNA steady state levels were significantly reduced in the leaf tip and mid leaves for maize and rice although not in sorghum (Fig. 4c,i). *CSE* mRNA levels were more uniform along the leaf gradient in all three species with slightly reduced levels in the leaf tips of maize and sorghum (Fig. 4d,j). Of the four phenylpropanoid targets genes examined, the *F5H* mRNA steady state profile differed in the three species. In maize, the lowest levels of *F5H* mRNA were observed in the leaf base and conversely at the tip in rice leaves, whereas a uniform low level was evident in sorghum (Fig. 4e,k). Thus, we found that *COMT1*, *4CL2*, and *CSE*, had similar developmental patterns of mRNA accumulation along the leaf in at least two species and were in general agreement with the transcriptomic analysis whereby phenylpropanoid target gene expression was generally reduced in the more mature mid and leaf tip regions (Figs 2 and 3). Exceptions to this included the maize *ZmF5H* gene which exhibited an opposite profile with highest mRNA levels in the leaf base instead of the tip (Fig. 4e,k), and high levels of *OsCOMT1* and *Sb4CL2* mRNA were not measured in the leaf base (Fig. 4b,c,h,i).

For *MYB31* and *MYB42*, there was also general agreement with the transcriptomic data and the pattern in sorghum was more similar to that in rice than maize (Fig. 4f–m). In maize there were low and uniform ZmMYB31 mRNA levels along the leaf gradient but for ZmMYB42 there were gradually increasing levels towards the leaf tip. In sorghum and rice, the highest level of *MYB31* mRNA was evident in the leaf base with levels dropping several fold in the middle and tip of the leaf (Fig. 4f,l,k), but there was not a significant change in *MYB42* mRNA levels along the leaf gradient for these two species (Fig. 4g,m).

Following the profiling of target gene and regulatory mRNA accumulation profiles we found that in some instances they could be correlated (see Supplementary Table S8). Previous work had suggested a negative role for MYB31 and MYB42 in the regulation of the *COMT* gene in maize^8^,^10^. Consistent with those studies, we did observe negative correlations between *ZmMYB42* mRNA levels and both *ZmCOMT1* (r = −0.98, p = 0.13) as well as *Zm4CL2* (r = −0.99, p = 0.08). This result would be anticipated if MYB42 acts as a negative regulator of these targets in more mature leaf tissues. However we observed a positive correlation between *MYB31* expression and *COMT1* in sorghum (r = 1.0, p = 0.05 and rice (r = 1.0, p = 0.02) which is inconsistent with previous *ZmMYB31* overexpression studies in Arabidopsis^8^.

In summary, it appears that although most of the tested phenylpropanoid and regulatory genes followed a general expression pattern aligned to the transcriptomic studies, there were significant deviations both within and between species. This underscores the importance of determining target gene expression in the same tissues used for promoter occupancy studies.

### Differential MYB31/MYB42 interactions with target phenylpropanoid genes in maize, sorghum and rice

**Figure 5:**
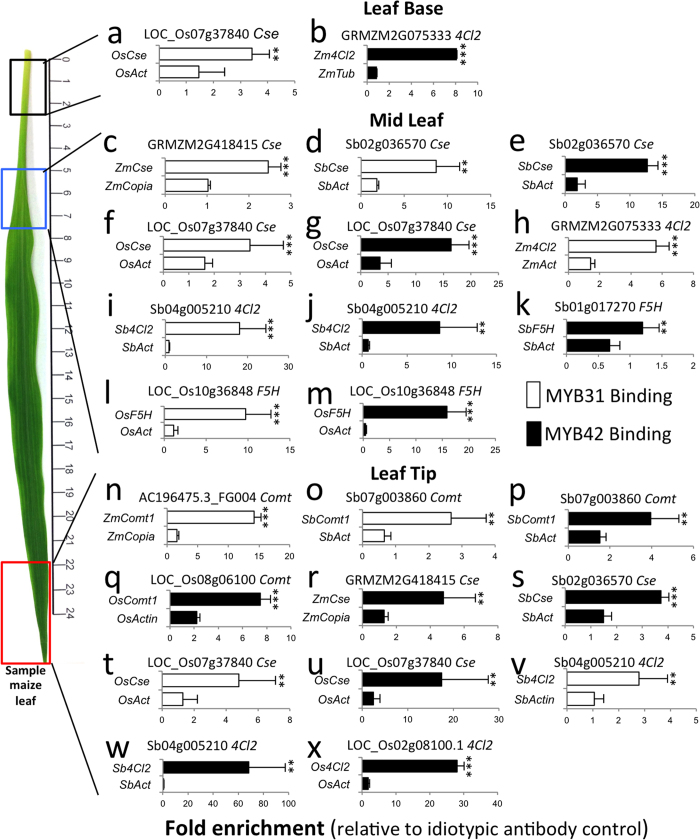
*In planta* binding of MYB31 and MYB42 to the promoter region of target phenylpropanoid genes in various regions of maize, sorghum and rice seedling leaves. (**a–x**) Fold enrichment of the binding of MYB31 (white bar graphs) and MYB42 (black bar graphs) relative to an idiotypic antibody control as determined by ChIP-PCR to the promoter region of select phenylpropanoid target genes in the leaf base (**a,b**), mid leaf (**c–m**) or leaf tip (**n–x**) tissues. The leaf image is of a typical maize leaf used in the analysis. Graphs represent one of three biological replicates with three technical repetitions (all data provided in Supplementary Table S8). Asterisks indicate statistically significant difference (Using Students *t*-test) in enrichment between sample and the actin, copia, or tubulin controls (**P ≤ 0.05, ***P ≤ 0.01, 1 tailed *t*-test). Enrichment is reported if observed in at least 2 of 3 biological replicates.

**Figure 6:**
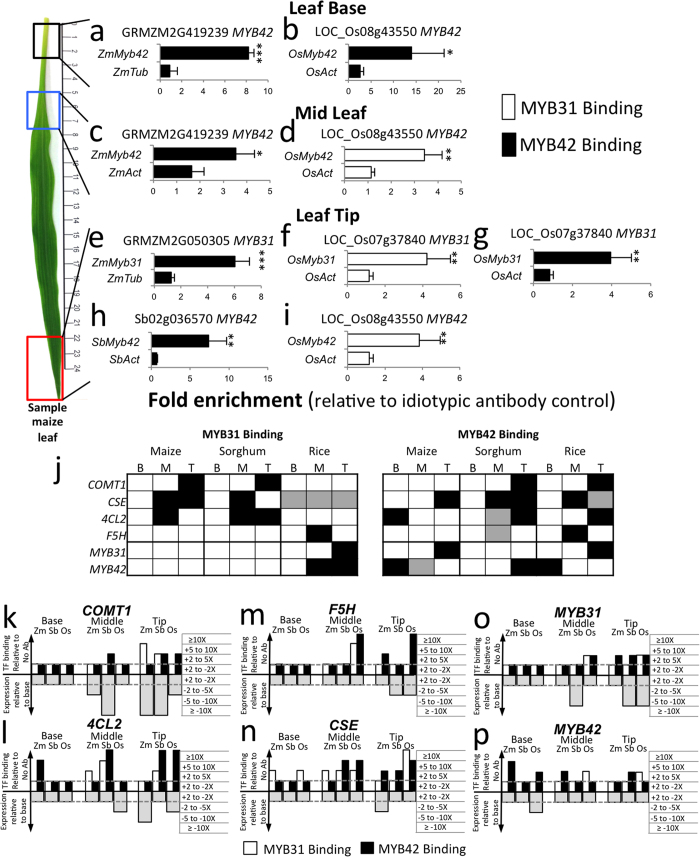
*In planta* binding of MYB31 and MYB42 to the promoter region of *Myb31* and *Myb42* regulatory genes in the leaf base, mid leaf, and leaf tip regions of maize, sorghum and rice seedling leaves. (**a–i**) Fold enrichment of the binding of MYB31 (white bar graphs) and MYB42 (black bar graphs) relative to an idiotypic antibody control as determined by ChIP-PCR to the promoter region of *MYB31* and *MYB42* regulatory target genes in the leaf base, mid leaf, and leaf tip regions of maize, sorghum and rice seedling leaves. Graphs are represented as in Fig. 5. (*P ≤ 0.1, **P ≤ 0.05, ***P ≤ 0.01, 1 tailed *t*-test). The leaf image is of a typical maize leaf used in the analysis. j: Summary of significant enrichments that detect MYB31 or MYB42 binding to the promoters of target genes in maize, sorghum, and rice in basal (B), mid leaf (M) and leaf tip (T) tissues (Figs 5 and 6 and see Supplementary Table S8. The p-values were obtained using Fisher’s combined probability test for significant enrichments and are reported in Supplementary Table S8, and color-coded as gray (P ≤ 0.05) or black (P ≤ 0.01) boxes. (**k–p**) Summary graphs of altered gene expression and promoter occupancy for *Comt*, *4CL2*, *F5H*, *Cse*, *MYB31* and *MYB42* respectively. The below line graphs represent average gene expression levels in mid and leaf tip tissues relative to the leaf base for three biological replicates. Results are binned in 4 categories (+2 to −2 fold, −2 to −5 fold, −5 to −10 fold and ≥−10 fold). The above line graphs represent the average of increased promoter occupancy by MYB31 (white boxes) or MYB42 (shaded boxes) relative to the no antibody control of which two or three biological replicates exhibited statistically increased binding relative to the control. Results are binned in 4 categories (+2 to −2 fold, −2 to −5 fold, −5 to −10 fold and ≥−10 fold).

To determine the MYB31/42 TFs promoter occupancy in phenylpropanoid gene targets *in planta*, we conducted ChIP-PCR experiments using specific antisera (see Supplementary Fig. 3Sa–c) in the same leaf sections used in gene expression analyses. Representative ChIP-PCR assay results are shown in Figs 5 and 6, a summary of MYB31 and MYB42 targets in Fig. 6j, and a complete table of ChIP-PCR results is provided in Supplementary Table S9. Summary graphs of promoter occupancy (Figs 5 and 6) and mRNA accumulation changes relative to base tissue (Fig. 4h–m) are provided in Figs 6k–p.

Two instances of phenylpropanoid gene promoter occupancy were detected in leaf basal tissue. Significant enrichment of MYB42 binding to the *Zm4Cl2* (P ≤ 0.01) and MYB31 to the *OsCse* (P ≤ 0.05) promoters was observed in maize and rice basal tissue (Fig. 5a,b). In contrast, a higher level of promoter occupancy by MYB31/42 was detected in the mid leaf tissue region of all three plant species where phenylpropanoid gene expression begins to decline. Notably, MYB31 binds the maize *CSE* promoter (P ≤ 0.01)(Fig. 5c), while in sorghum and rice the same promoter was recognized by both MYB31 and MYB42 (Fig. 5d–g). For the *4CL2* gene which functions in the early part of the pathway, MYB31 was found binding to the maize *Zm4Cl2* promoter (Fig. 5h), whereas both MYB31 (P ≤ 0.01) and MYB42 (P ≤ 0.05) were found to bind to sorghum *Sb4Cl2* (Fig. 5i,j). In the latter stage of the pathway, *F5H* was occupied by MYB42 in sorghum (P ≤ 0.1)(Fig. 5k), and by both MYB31 (P ≤ 0.01) and MYB42 (P ≤ 0.01) in rice (Fig. 5l,m).

A greater level of target phenylpropanoid gene promoter occupancy by MYB31 and MYB42 was also detected in the leaf tip tissues. Interestingly, the *COMT* promoter was occupied in all three species, by MYB31 in maize (P ≤ 0.01), by MYB42 in rice ((P ≤ 0.01), and by both MYB31 (P ≤ 0.05), and MYB42 (P ≤ 0.05) in sorghum (Fig. 5n–q). The *CSE* promoter was again found to be occupied by MYB31 or MYB42 in maize and sorghum and by both TFs in rice (Fig. 5r–u). Lastly, MYB31 occupied the *4CL2* promoter in sorghum and both MYB31 and MYB42 occupied the *4Cl2* promoter in rice (Fig. 5v–x).

Overall MYB31 and MYB42 seem to occupy phenylpropanoid promoters in the more mature mid (n = 11, 44%) and leaf tip (n = 12, 48%) tissues of all three species with few instances of enriched binding (n = 2, 8%) detected in the young basal tissue (Fig. 6k–p). Together, these findings provide insights into the dynamics of MYB31 and MYB42 binding activity along the leaf developmental gradient and show that MYB31 and MYB42 in addition to binding *COMT1*, also occupy *4CL2*, *F5H*, and *CSE* promoters in maize, sorghum and rice.

### Differential MYB31/MYB42 interactions with regulatory genes in maize, sorghum and rice

*ZmMYB31* and *ZmMYB42* contain one or three ACII elements respectively and bind to their own promoters in maize seedling leaf tissue^26^. To further understand the roles of MYB31 and MYB42 in the regulatory network governing leaf differentiation, we evaluated the binding of these TFs to the their own gene promoters in maize, sorghum, and rice. We found that MYB42 binds to its own promoter in maize and rice basal leaf tissue (P ≤ 0.05, P ≤ 0.1, respectively) (Fig. 6a,b) and in the leaf tip tissue of sorghum (P ≤ 0.05) (Fig. 6h). MYB42 also bound to the MYB31 promoter in the leaf tip tissue of maize and rice (P ≤ 0.05) (Fig. 6e,g). In contrast, MYB31 bound to its own promoter in rice leaf tip tissue (P ≤ 0.05)(Fig. 6f), and to the *OsMYB42* promoter in both mid leaf and leaf tip tissues (P ≤ 0.05) (Fig. 6d,i). In summary, our results indicate that MYB42 is involved in autoregulation in all three species and cross occupancy of *MYB31* in maize and rice (Fig. 6k–p). Evidence for MYB31 autoregulation and cross occupancy was observed only in rice.

### Control of transcription of phenylpropanoid genes by ZmMYB31 and ZmMYB42

**Figure 7:**
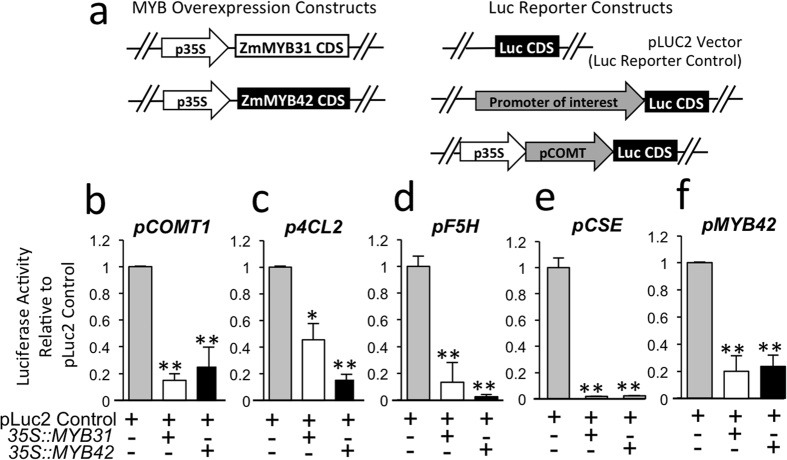
Down-regulation of *Zm*C*OMT1*, *Zm4Cl2*, *ZmF5H*, *ZmCSE*, and *ZmMYB42* genes by ZmMYB31 and ZmMYB42 *in vivo.* (**a**) Schematics of overexpression, and reporter constructs used to test ZmMYB31 and ZmMYB42 regulatory activity in maize protoplast assays. *ZmMYB31* and *ZmMYB42* coding sequences were cloned into vectors in which the 35S promoter drives overexpression^8^. The empty pLUC2 vector serves as the reference control for luciferase activity. Promoters of interest are cloned upstream of the LUC2 CDS in reporter constructs except for ZmCOMT1 which included a 35S enhancer to boost expression levels^8^. (**b–f)** Protoplast Reporter assays for *ZmCOMT1* (**b**), *Zm4Cl2* (**c**), *ZmF5H* (**d**), *ZmCSE* (**e**) and *ZmMYB42* (**f**). Reporter assays were performed either in the presence of plasmids overexpressing *ZmMYB31* (white) or *ZmMYB42* (black). Expression level is reported as luciferase activity relative to the pLUC2 empty vector alone (gray) and represents the average of four independent transformations with three technical replicates each. Asterisks indicate statistically significant difference (using Student’s *t*-test) in enrichment between sample and the reporter construct alone control (*P ≤ 0.05, **P ≤ 0.01, 2 tailed *t*-test).

The enrichment of MYB31 and MYB42 binding to phenylpropanoid target promoters and their own promoters in some instances correlated with a reduction in mRNA accumulation relative to the leaf base (Fig. 5j–p). For example, enriched binding of ZmMYB31 to *ZmCOMT1*occurs in maize leaf tips (Fig. 6j,k) and enriched binding of OsMYB42 to *Os4CL2* in rice leaf tips (Fig. 6j,l). However in other instances enriched binding does not appear to correlate with reduced gene expression *e.g.* enriched binding of ZmMYB42 to *ZmF5H* in maize mid leaf tissue (Fig. 6j,m) and enriched binding of SbMYB42 to *Sb4CL2* in sorghum mid leaf tissue (Fig. j,l). To reveal whether MYB31 and MYB42 function as activators or repressors *in vivo*, we performed transient expression assays in maize, as previously described^26^. A ~1 kb promoter region from the transcriptional start site (TSS) of the maize *COMT1*, *4CL2*, *F5H* or *CSE* genes was cloned upstream of the luciferase reporter gene to assay the effect of ZmMYB31 and ZmMYB42 on their expression. ZmMYB31 and ZmMYB42 overexpression constructs (Fig. 7a), driven by the constitutive *CaMV 35S* promoter were electroporated into maize protoplasts along with each of the promoter::luciferase reporters (Fig. 7a). We found that both ZmMYB31 and ZmMYB42 decreased by 25% (p < 0.01) the expression of the pCOMT::Luc reporter, as compared with control (Fig. 7b), in agreement with previous studies^8^. Our results showed that ZmMYB31 and ZmMYB42 also significantly (p < 0.01) inhibited *Zm4Cl2*, (p < 0.01), *ZmCSE* (p < 0.05, p < 0.01), and *ZmF5H* (p < 0.01) luciferase expression (Fig. 7c–e). To further understand the cross regulatory regulation of MYB42/31, we asked if expression of a pMYB42::Luc reporter construct was affected by MYB31 or MYB42. Our results showed that MYB42-luciferase expression was significantly downregulated by both ZmMYB31 and ZmMYB42 itself (Fig. 7f) supporting a cross regulatory mechanism. In summary, ZmMYB31 and ZmMYB42 act as repressors on all of the reporter constructs tested, indicating that these MYB factors can act predominantly as negative regulators of phenylpropanoid targets *in vivo*.

## Discussion

In monocot leaves there is a primary gradient of metabolic activities that switch from photosynthetic sink to source from the youngest dividing tissue at the leaf base to the mature differentiated tissue at the leaf tip. During leaf development phenylpropanoid compound production is required in the leaf base where leaf xylogenesis occurs with concomitant lignification of cell walls and this process is complete around the ligule region of the leaf^27^,^28^. Both positive and negative regulatory TFs play a role in the regulation of phenylpropanoid biosynthetic genes along this developmental gradient^8^,^10^,^18^,^24^,^29^. Although MYB31 and MYB42 have been implicated in negatively regulating lignin content in maize, their activities along the leaf development stages and their target repertoires in other monocot leaves have not been assessed. Here, we determined the regulatory dynamics of these MYB factors along the leaf developmental gradient in three monocot species and uncovered differential activity that is easily overlooked if entire leaves are analyzed^25^,^30^,^31^. Our results also point to subfunctionalization of the roles of these MYBs in monocots following WGD events and within a regulatory network that also involves recently identified MYB TFs that are activators of phenylpropanoid biosynthetic genes.

**Figure 8:**
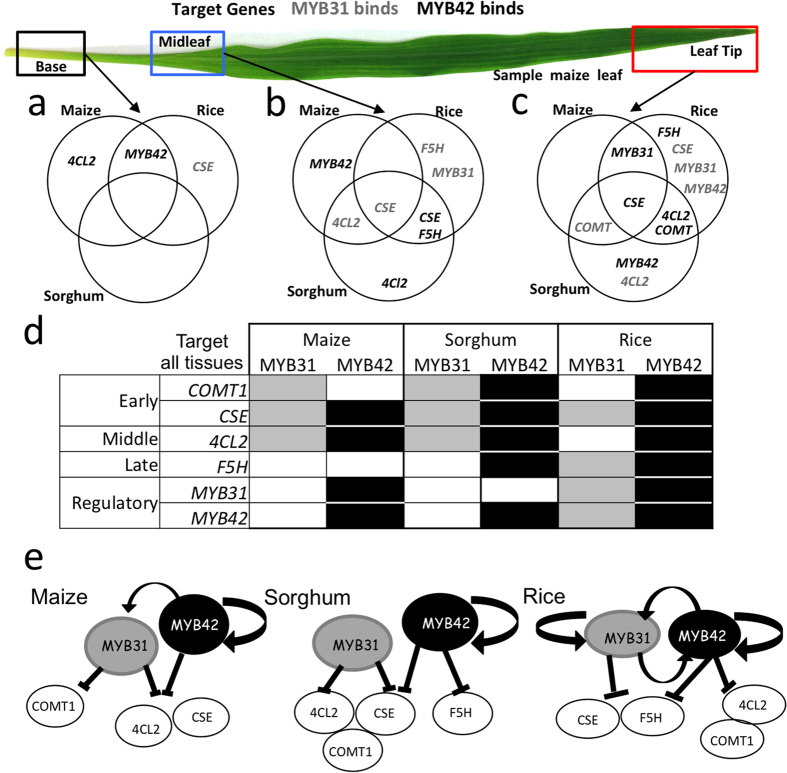
Phenylpropanoid and regulatory gene promoter occupancy by MYB31 and MYB42 and proposed regulatory modules in maize, sorghum and rice seedling leaf tissues. (**a–c**) Venn diagrams of promoters that exhibited >2 fold and statistically significant enrichment of MYB31 (gray text) or MYB42 (black text) binding in leaf basal tissue (**a**), mid leaf tissue (**b**), or leaf tip tissue (**c**) for all three species as determined by ChIP-PCR in at least 2 of 3 biological replicate experiments. The leaf image is of a typical maize leaf used in the analysis. (**d**) Tabular summary of enriched MYB31 (Gray shading) and MYB42 (black shading) binding to *COMT1, CSE, 4CL2*, and *F5H* phenylpropanoid gene and *MYB31/42* regulatory targets all tissues (base, mid and leaf tip) of maize, sorghum and rice. (**e**) Proposed models for regulatory and autoregulatory roles of MYB31 and MYB42 in maize, sorghum, and rice leaves.

In support of the proposed role of MYB31 and MYB42 as negative regulators of phenylpropanoid genes our results showed that 46% of the available regulatory space was occupied by MYB31 and MYB42 in the leaf tip and mid leaf regions as opposed to 8% in the leaf base (Figs 6j–p and 8a–d). This pattern of increased binding coincides with the observed general reduction phenylpropanoid gene expression beginning near the leaf ligule and beyond (Figs 2, 3, 4 and Supp. Fig. S4)^25^,^30^. Indeed our co-expression and qRT-PCR analysis revealed negative correlations between *MYB42* mRNA levels and those of *COMT1* in sorghum and both *COMT1* and *4Cl2* in maize (Supplementary Table S7). Such a reduction in phenylpropanoid gene expression could be explained by increased promoter binding by negative regulators^8^,^9^,^10^,^12^. For example, a reduction in expression of the *COMT1* gene towards the leaf tip was accompanied by increased occupancy by MYB31 (in maize and sorghum) or by MYB42 (in sorghum and rice). In rice, binding by both MYB31 and MYB42 to *F5H* in mid leaf tissue was accompanied by a several fold reduction in gene expression. Conversely, the low occupancy of phenylpropanoid promoters in the leaf base correlated with the highest level of expression of many of the genes in this pathway. In maize the inverse correlation between MYB38/42 expression and expression of several structural genes also supports a possible role for these as negative regulators. Together these observations support the hypothesis that MYB31 and MYB42 are acting as negative regulators *in planta* in each of these grasses. The fact that their binding activities differ along the leaf developmental gradient underscores the importance of conducting these studies with finer tissue and cellular resolution.

The regulation of phenylpropanoid gene expression involves both positive and negative regulators of which only a few have yet been described. A complete description of the regulatory network governing phenylpropanoid metabolism will require understanding the dynamic relationship between these regulators. In several instances, our study found that enhanced binding by MYB31 or MYB42 was either not accompanied by a change in gene expression, or a change in gene expression was not accompanied by enhanced binding. For example, in maize, the expression *of Zm4CL2* was negatively correlated with *ZmMYB42* expression along the leaf gradient, but MYB42 binding was detected in the leaf base where *Zm4CL2* expression was highest. Also, there was no correlation between the relatively uniform expression of *CSE* expression along the leaf gradient and its frequent targeting by MYB31 or MYB42 in all three species. The phenomenon of promoter occupancy without an obvious effect on target gene expression suggests that co-regulators or post-translational phosphorylation may be involved in modifying MYB31/42 function^32^,^33^,^34^,^35^,^36^. To date the repressor activities of MYB31 and MYB42 have been largely inferred from overexpression studies in plants^9^,^10^ or as we and others have shown, by their negative effect on reporter constructs during transient expression in protoplasts^8^,^12^,^26^. It is possible that MYB31 and MYB42 do not act only in a repressive manner *in planta*. Alternatively, MYB31 and MYB42 may generally act as repressors but compete with gene activators to achieve a tissue specific level of target gene expression. For example two instances of enriched binding of OsMYB31 and ZmMYB42 binding to *OsCSE* and *Zm4Cl2* respectively were observed in basal leaf tissues although the expression level of these genes was high. In these basal tissues OsMYB31 and ZmMYB42 are expressed and the latter appears to participate in auto-regulation in basal tissue. In these instances, the binding by MYB31/42 regulators to phenylpropanoid targets may be necessary to achieve the correct tissue specific expression level by competing with activators of the phenylpropanoid pathway. Indeed two recent studies have provided evidence that ZmMYB5 and ZmMYB152 and SbMYB60 act as positive regulators of phenylpropanoid metabolism in maize and sorghum respectively^18^,^24^. Our analysis reveals that the syntelogs of these genes in all three species are co-expressed, albeit at low levels, with phenylpropanoid genes in the basal leaf area (Figs 2 and 3 and Supp. Fig. S4). For example the *ZmMYB152, SbMYB22*, and *OsMYB107* syntelogs exhibit a peak expression in the basal leaf area, coinciding with maximal phenylpropanoid biosynthetic gene expression, and then are almost undetectable in more mature tissues where phenylpropanoid gene expression is lowest. Thus it is appears that several R2R3 MYB TFs that act in a positive and negative manner are competing for binding and thus may explain why promoter occupancy by MYB31 and or MYB42 in this study was not always clearly correlated with reduced target gene expression. Future co-occupancy, post-translational modification, and protein turnover^12^ studies in specific plant tissues will be required to further dissect how the balance between these positive and negative MYB TFs results in fine-tuning of the phenylpropanoid gene pathway.

The comparison of maize, sorghum, and rice which differ in their number of MYB31/42 ohnologs provided an opportunity to investigate the consequences of regulatory network evolution following WGD events^37^,^38^,^39^,^40^,^41^,^42^. In general the fate of duplicated genes following WGD is poorly understood but an RNA-seq study of 18,000 duplicated genes in soybean revealed that about half of all genes and especially TFs were differentially expressed and had undergone expression sub-functionalization^40^. Our survey of promoter occupancy in different leaf tissues in three species found that only about one fifth of MYB31 or MYB42 binding events were detected in two or more species (Fig. 8a–d). The fact that *COMT1* and *CSE* were commonly targeted by both MYBS in all three species suggests that MYB31 and MYB42 are part of an ancient regulatory module in leaves. The fact that the pattern of binding varies across tissues and species (Fig. 8e) suggests that this ancient module is undergoing evolutionary change. Indeed the gene regulatory models that can be built by our findings (Fig. 8e) suggest a rapid divergence of gene regulation following speciation and genome duplication. Tissue-specific targets that were shared between at least two species may represent parts of the regulatory network that have not changed since the species diverged from a common ancestor^41^. For example in sorghum *SbCOMT* and *Sb4CL2* are targeted by SbMYB31 and SbMYB42 but in maize *ZmCOMT* targeting by ZmMYB42 was not detected and in rice both *OsCOMT* and *Os4CL2* targeting by OsMYB31 was not detected (Fig. 8d,e). Targeting of both genes may have been the ancestral situation with subfunctionalization occurring following the WGD event. Future studies will help to pinpoint the precise elements targeted by the MYB31 and MYB42 proteins in the promoter regions of the target genes identified in this study and how *cis*-element changes may have contributed to the observed differential targeting^43^.

Our results suggest that auto-regulation and cross-regulation of MYB31 and MYB42 expression also occurs *in planta*. We found that *MYB42* is a target of MYB42 in the basal leaf tissue of maize and rice, the mid leaf region of maize (Fig. 8c), and in the leaf tip region of sorghum (Fig. 8a–c), suggesting auto-regulation in these tissues. This existence of this autoregulatory loop in all three species suggests that this regulatory module may have predated the speciation of these grasses (Fig. 8e–g), but which is now undergoing change in a tissue specific manner. MYB42 also binds the promoter of the *MYB31* gene in the leaf tip of maize and rice (Figs 6j and 8d). In the latter case, MYB42 binding correlated with reduced expression of *MYB31* in the leaf tip of rice suggesting cross regulation. In rice, there was evidence for feedback regulation as *OsMYB31* and *OsMYB42* are both targeted by MYB31 and *OsMYB31* is targeted by MYB42 in both mid leaf and leaf tip tissues. The existence of an extra autoregulation loop in rice might be explained when it is considered that MYB31 and MYB42 are ohnologs that arose as a result of the ancient WGD event that preceded monocot divergence. If autoregulation of the MYB31/42 ancestral gene existed prior to the ancient WGD then it may have been retained by both ohnologs in the rice lineage but lost for MYB31 in the sorghum/maize lineage. In maize it is also likely that the ZmMYB11 and ZmMYB38 ohnologs participate in this autoregulation. In this context it will be of interest to determine if these autoregulatory loops have been retained in other monocot species. Furthermore the observed co-expression of both positive and negative MYB TFs leads to the possibility that MYB5/13/19/152 fall within the domain of the observed MYB31/42 cross-regulation and the possibility of indirect down-regulation of phenylpropanoid targets.

In summary, we demonstrate that syntelogs of MYB31 and MYB42 bind to phenylpropanoid genes that function in the early, mid and late stages of the pathway in three monocot grasses. Some conservation of regulatory targets such as *COMT1*, *CSE* and autoregulation of MYB42 is suggestive of an ancient regulatory module. We mostly found however that there is divergence of MYB31/42 target repertoires across species and tissues. These changes likely reflect a sub-functionalization of the regulatory genes following gene duplication. In several instances the occupancy of phenylpropanoid promoters by these MYB regulators is correlated with reduced expression of the target genes and this is consistent with evidence that these act as negative regulators *in vivo*. The fact that promoter occupancy usually is not correlated with changes in gene expression is suggestive of a possible role for co-regulators, posttranslational modification, and activator TFs in modifying MYB31/42 activity. Further elucidation of the nuances of MYB31/42 regulatory functions may prove helpful in the engineering of the flux of lignin metabolites in monocots being used as renewable biofuel sources^44^,^45^,^46^.

## Materials and Methods

### Plant Material and Sample Collection

Maize (*Zea mays* inbred B73), sorghum (*Sorghum bicolor*, BTx623) and rice (O*ryza sativa* cv. Nipponbare) were grown in a Conviron E7/2 growth chamber under a 28/25 °C temperature and 12 hr light (~300 μE)/dark regime. Plants were grown in a commercial soil mix, (Promix BX Mycorrhizae, Premier Tech, Quakertown, PA) supplemented with 48 g of lime (Pennington Fast Acting lime, Pennington Fertilizer, Inc., Atlanta GA) and 20 g of slow release fertilizer (Scotts 18-6-12 Osmocote Classic, The Scotts Co., Maryland, OH) per 28 L of soil. For gene expression and *in vivo* binding studies maize, sorghum, and rice seedlings were grown for 9, 16, and 20 days post germination, respectively. The relevant leaf tissue sample was harvested and then frozen in liquid nitrogen either immediately or after cross-linking. All samples were collected 3 hours after turning lights on to minimize circadian rhythm effects. For protoplast experiments, maize B73 × Mo17 hybrid seeds were employed. Seeds were germinated, then sown in the soil mix described above, and grown in complete dark for 9 days prior to protoplast isolation (see below for details).

### Quantitative measurement of gene expression by RT-qPCR

Three biological replicate samples from the leaf base, middle and tip tissues for each species were harvested, frozen immediately in liquid nitrogen, and stored at −80 °C. Total RNA was extracted using the PureLink Plant RNA reagent (ThermoFisher Scientific Waltham, MA). DNA was removed from RNA samples using the Turbo-DNA-*free* kit (ThermoFisher Scientific Waltham, MA). RNA concentration was determined using a Nanodrop 1000 spectrophotometer and quality checked by examination on formaldehyde gels. 6 ng of RNA was used in performing RT-qPCR reactions with the Verso 1-step RT-qPCR Kit, (SYBR Green, ROX) (ThermoFisher Scientific, Waltham, MA) on a Bio-Rad CFX96 Real-Time PCR Detection System. Gene-specific primers were designed using the primer3 (v. 0.4.0) software, (www.bioinfo.ut.ee/primer3-0.4.0/). Specificity of the RT-PCR reaction was determined by performing a melting curve for every sample as well as no-RT and no-template controls. The expression levels of the genes of interest were normalized to a constitutive gene namely tubulin in maize, ubiquitin in sorghum and rice (see Supplementary Table S1). Normalization was performed by subtracting the cycle threshold (CT) value of the constitutive gene from the CT value of the gene of interest (ΔCT). The expression level in each tissue was determined for three biological replicates with three technical replicates each. The values (n = 9) were plotted using a boxplot^47^.

### *In silico* expression and coexpression analyses

The gradient of expression of phenylpropanoid and MYB regulatory genes in maize and rice seedling leaves was analyzed using previously published RNA-seq data^24^,^25^. The RPKM values for the selected genes were retrieved, and the values for 15 developmental leaf sections in maize, 13 in sorghum, and 11 in rice were normalized by transforming to log2 RPKMs. A heat-map of the tissue-specific expression pattern was established by average clustering analysis, with Euclidean distance, and single linkage distance between clusters, was calculated in the R software environment. The Spearmen’s correlation coefficient was calculated using the Rcorr function in the ‘Hmisc’ package^48^.

### Cloning, overexpression, and purification of recombinant proteins

The regions encoding amino acids 128–194 (67aa) of SbMYB31 and 135–202 (68aa) of SbMYB42, 128–186 (59aa) of OsMYB31 and 126–186 (61aa) of OsMYB42 were amplified using PCR and the primer pairs (SbMYB31Uniup and SbMYB31Unidn), (SbMYB42Uniup and SbMYB42Unidn), (OsMYB31Uniup, and OsMYB31Unidn), and (OsMYB42Uniup and OsMYB42Unidn) respectively (Fig. 1 and see Supplementary Table S1). PCR reactions utilized a high fidelity polymerase (Phusion HF polymerase, New England Biolabs Inc., Ipswich MA). PCR products were column purified and cloned into the pENTR-SD/TOPO vector (Invitrogen, Carlsbad CA) to create the entry clones pSbMYB31Uni-D/TOPO, pSbMYB42Uni-D/TOPO, pOsMYB31Uni-D/TOPO, pOsMYB42Uni-D/TOPO respectively (Table 2). Clones were confirmed to be devoid of amplification errors by Sanger dideoxy sequencing. Entry clones were recombined with pDEST15 and pDEST17 using LR Clonase (Invitrogen, Carlsbad, CA) to create expression clones with an N-terminal GST tag or 6xHis tag respectively (S2 Table). The plasmids were transformed into *E. coli* BL21-DE3 cells and expression of the fusion proteins induced by the addition of 1 mM isopropyl-beta-D-thiogalactopyranoside (IPTG) to cell cultures at an OD_600_ between 0.5–0.6. Following incubation for 3 hr at 30 °C the cells were harvested by centrifugation and 6xHis tagged proteins were purified using a His-Bind purification kit (EMD Millipore, Merck KGaA, Darmstadt, Germany). Elution fractions were analyzed using 12% SDS-PAGE, and stained with Coomassie Brilliant Blue R-250. PAGE gel slices containing at least 500 μg of protein were excised and used for antibody production.

### Polyclonal antibody production and purification

Unique regions of the SmMYB31, SbMYB42, OsMYB31 and OsMYB42 were overexpressed as described above and used to generate polyclonal antibodies in rabbits (Cocalico Biologicals, Inc., Reamstown, PA). To reduce cross-reactivity with unrelated proteins the polyclonal antiserum was further purified by affinity purification against GST tagged proteins following the method previously described^26^,^49^. The specificity of the purified antiserum was tested by western blot analysis as previously described^26^ except that a 1:250 dilution of polyclonal antiserum was used.

### Chromatin immunoprecipitation (ChIP) methodology

Pooled leaf sections were collected from 10–15 soil-grown maize, sorghum or rice seedlings (Fig. 1c) and cross-linking with formaldehyde performed immediately. ChIP experiments were performed as described previously^50^ with a few modifications described below. Following grinding the frozen tissue was re-suspended in 1 ml of NIB buffer instead of 0.5 ml. Later 450 μl of lysis buffer was added instead of 400 μl and sonication was performed on ice using a UCD-200 model Biorupter sonicator (Diagenode Inc., Denville, NJ). The setting was at power H for a total of 30, 20 and 15 min for maize, sorghum, and rice respectively employing a 30 sec on 30 sec off cycle and ice was changed every 10 min. After the pre-clearing step, immunoprecipitation was performed with 1 ul of purified antisera. For enrichment tests on ChIPed material, gene specific primers for target genes (*COMT1, CSE, 4Cl2, F5H, MYB31*, and *MYB42*) and for a non-bound control (Actin or Tubulin) were used in PCR reactions with immunoprecipitated and input DNA for each plant species. The primer sequences are listed in S2 Table. Quantitative (real time) PCR (qPCR) was performed using the iQ SYBR Green supermix (Bio-Rad, Hercules, CA) in a 20 μl qPCR reaction. Specifically, 1 μl of recovered DNA from ChIP, idiotypic antibody control, or input DNA was added to a reaction consisting of 10 μl iQ SYBR Green supermix and 0.25 μM of each primer in a 20 ul reaction. PCR was performed using a BioRad CFX96 real-time PCR Detection System (Applied Biosystems, Foster City, USA) with an initial 95 °C, 3 min denaturation followed by 40 cycles of 95 °C, 10 s; 60 °C, 15 s; 72 °C, 30 s, followed by a melting curve determination. With all experiments, idiotypic antibody controls, and input sample controls were performed for every primer set used. Quantitation involved normalization of each immune precipitation (or control, idiotypic antibody)^8^.

### Generation of constructs for transient expression experiments

Previously described plant expression vectors include, p35S::ZmMYB31 (pUT2302)^8^, and p35S::ZmMYB42^9^. The p35S::BAR (pPHP611) plasmid was used for normalizing the concentration of p35S sequences delivered in each electroporation^51^. Reporter constructs were generated for maize genes by amplifying the promoter region using B73 genomic DNA as template. The *4Cl2* (0.96 kb), *F5H* (0.91 kb), *COMT1* (0.812 kb) and *CSE* (0.739 kb) promoter regions upstream of the mapped TSS sites^52^ were PCR amplified using specific primer pairs Zm4Cl2promF1 and Zm4Cl2promR1, ZmF5HpromF1, and ZmF5HpromR1, ZmCOMT1promF1 and ZmCOMT1promR1, ZmCSEpromF1and ZmCSEpromR1, respectively (S3 Table). The PCR products were first cloned into the pENTR/SD/D-Topo entry vector (Invitrogen, Carlsbad, CA), and then recombined with the pLUC2 luciferase vector via LR recombination to create p4Cl2::Luc, pF5H::Luc, pCOMT1::Luc, p1CSE::Luc, p2CSE::Luc respectively. In the case of the *ZmCOMT1* reporter construct a CaMV 35S enhancer was included upstream of the COMT1 promoter region (Fig. 7a) to ensure measurable luciferase levels because previous work had shown that the basal transcription of p*COMT* is so low in protoplasts that an inhibitory effect is undetectable^26^. The addition of a CaMV 35S enhancer did not prove necessary to detect an inhibitory effect on the other promoters.

### Transient expression assays in maize protoplasts

Transient expression assays in maize protoplasts (B73 × Mo17) were performed as described previously with minor modifications^53^. For the protoplast isolation buffer, 3% (w/v) cellulase “Onuzuka” R-10 (bioWorld, Dublin, OH) and 0.6% (w/v) macerozyme (Desert Biologicals, Phoeniz, AZ) were employed. Electroporation was performed using ~10^5^ protoplasts with a total of 45ugs DNA per transformation (total volume 300 ul). Samples were given two 0.5–1 kV/cm for 10 msec pulses on a Bio-Rad Gene Pulser Electroporator. 35S::Renilla was used as a normalization control in all bombardments. Firefly luciferase and Renilla luciferase activity was assayed using the Dual-Luciferase Reporter Assay System (Promega, Madison, WI) on a Centro LB960 luminometer (Berthold Technologies). Each treatment was performed for at least 4 times with three technical replicates each. The luminescence values were normalized against the Renilla control, and the fold gene activation was calculated as the ratio between each particular treatment (plus MYB expression construct) and the control treatment (reporter fusion alone).

## Additional Information

**How to cite this article**: Agarwal, T. *et al*. MYB31/MYB42 Syntelogs Exhibit Divergent Regulation of Phenylpropanoid Genes in Maize, Sorghum and Rice. *Sci. Rep.* **6**, 28502; doi: 10.1038/srep28502 (2016).

## Supplementary Material

Supplementary Information
