# Supplementary material for: MYB31/MYB42 Syntelogs Exhibit Divergent Regulation of Phenylpropanoid Genes in Maize, Sorghum and Rice
Source: Sci Rep. 2016 Jun 22;6:28502. doi: 10.1038/srep28502 (PMC4916418; doi:10.1038/srep28502)
Supplement: Supplementary Information [file srep28502-s1.pdf]

MYB31/MYB42 Syntelogs Exhibit Divergent Regulation of Phenylpropanoid Genes in Maize, Sorghum and Rice

Tina Agarwal, Erich Grotewold, Andrea I. Doseff, and John Gray.

Supplemental Figures and Tables:

Supplemental Figure 1: Identification of ZmMYB31 and ZmMYB42 syntelogs in sorghum and rice. a-d: 2D synteny map views illustrating the length of the syntenic region and the extent of conservation of gene order between ZmMYB31 (GRMZM2G050305) (**a**), ZmMYB11 (GRMZM2G000818) (**b**), ZmMYB42 (GRMZM2G419239) (**c**), ZmMYB38 (GRMZM2G084583) (**d**), and their homologous genes (Sb02g031190.1, LOC_Os09g36730), (Sb02g031190, LOC_Os09g36730), (Sb07g024890, LOC_Os08g43550), and (Sb07g024890, LOC_Os08g43550) in sorghum and rice respectively. The strength of synteny in each region is indicated by block scores.

Supplemental Figure 2: Identification of ZmMYB5, ZmMYB13, ZmMYB19, and ZmMYB152 syntelogs in sorghum and rice. a-d: 2D synteny map views illustrating the length of the syntenic region and the extent of conservation of gene order between ZmMYB13 (GRMZM2G038722) (**a**), ZmMYB5 (GRMZM2G097636) (**b**), ZmMYB19 (GRMZM5G833253) (**c**), ZmMYB152 (GRMZM2G104551) (**d**), and their homologous genes (Sb06g027180, LOC_Os04g50770), (Sb06g027180,

LOC_Os04g50770), (Sb04g031110, LOC_Os02g46780), and (Sb02g030900, LOC_Os09g36250) in sorghum and rice respectively. The strength of synteny in each region is indicated by block scores.

Supplemental Figure 3: Generation of specific antisera to recognize MYB31 and MYB42 syntelogs in maize, sorghum and rice. a: Multiple sequence alignment of the regions of MYB31 and MYB42 and their homologs that were used to generate TF specific antiserum. Regions highlighted in grey indicate regions subcloned and overexpressed for antiserum generation. A conserved Ethylene-responsive element binding factor-associated amphiphilic repression (EAR) motif is highlighted in red. **b:** Western blot analysis indicating specificities of antisera generated against MYB31 and MYB42 proteins from maize, sorghum and rice. Images on the left panel are Coomassie blue stained PAGE gel of fractions of nickel affinity purified 6xHis-ZmMYB31 and 6xHisMYB42 fragments expressed in *E. coli*, that were used in western blot analysis. Arrows indicate sizes of standard (std) proteins. The middle and right panels represent western blots using either anti-MYB31 or anti-MYB42 antisera respectively. **c:** Top Panel shows Coomassie blue stained PAGE gel of fractions of nickel affinity purified 6xHis-ZmMYB11 and 6xHisMYB38 expressed in *E. coli*. Arrows indicate correct sized bands of 30kD and 32kD for 6xHis-ZmMYB11 and 6xHisMYB38 proteins in Induced clear lysate (I-CL) and eluate (E) fractions but not in uninduced cleared lysate (UI-CL) fractions. Std = Protein molecular weight standards. Lower panel shows Western dotblot analysis of fractions in upper panel. Left lower panel shows cross reaction of affinity purified

anti-ZmMYB31 with ZmMYB31 control but not fractions (E1) containing ZmMYB11 protein. Right lower panel shows cross reaction of affinity purified anti-ZmMYB42 with ZmMYB42 control but not fractions (E1) containing ZmMYB138 protein.

Supplemental Figure 4: Expression profiles of MYB regulatory genes and their phenylpropanoid targets in maize, sorghum and rice leaves. The average gene expression level (FPKM) versus leaf segment in **a**: maize, **b:** sorghum and **c:** rice. Leaf segments are numbered from leaf base to leaf tip. The RNA-Seq data for maize and rice were obtained from Wang *et al.,* 2014 and for sorghum from Ding *et al.,* 2015 respectively.

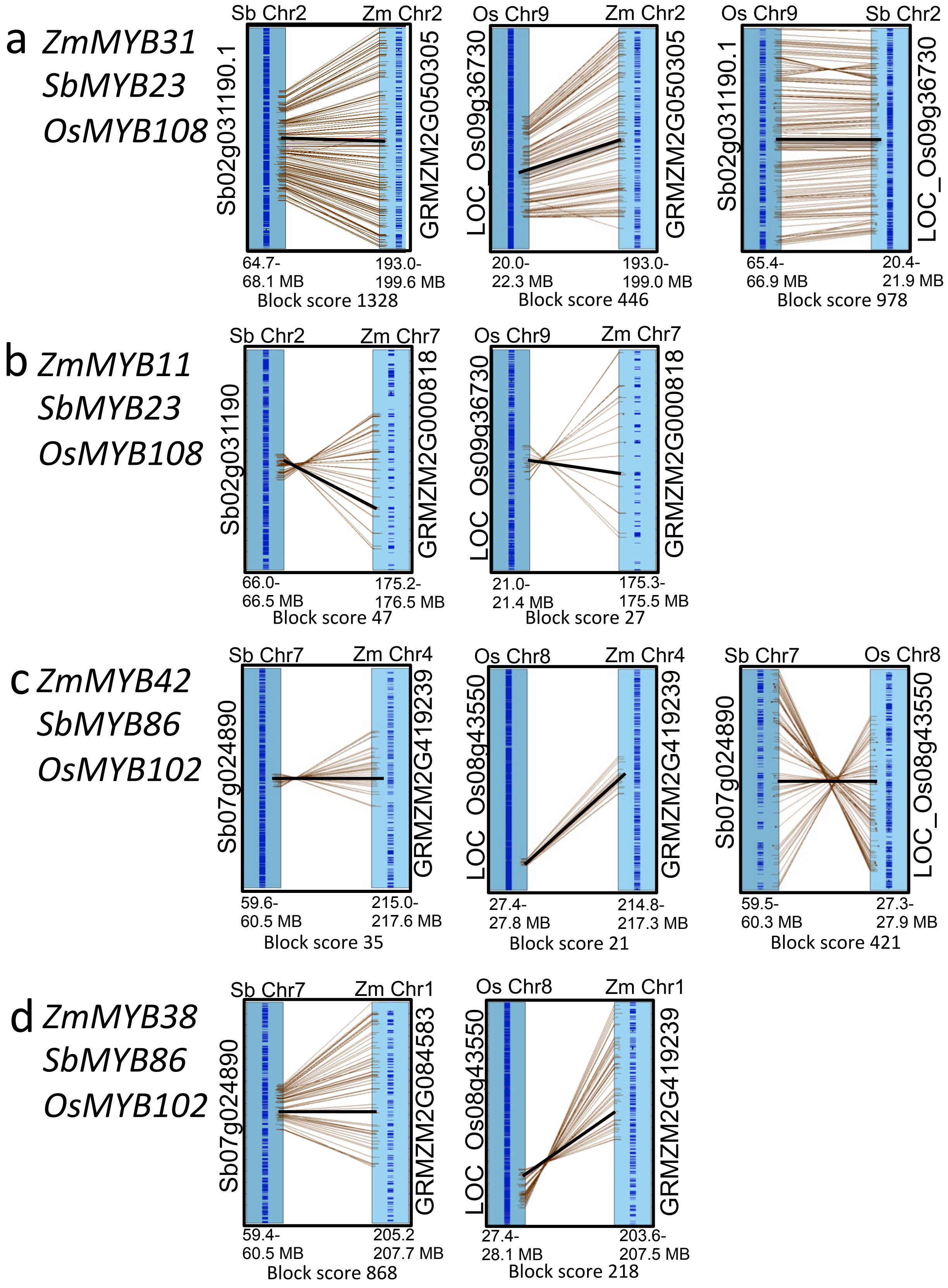
a ZmMYB31
SbMYB23
OsMYB108
Sb Chr2
Zm Chr2
Sb02g031190.1
GRMZM2G050305
64.7-
68.1 MB
193.0-
199.6 MB
Block score 1328
Os Chr9
Zm Chr2
LOC_Os09g36730
GRMZM2G050305
20.0-
22.3 MB
193.0-
199.0 MB
Block score 446
Os Chr9
Sb Chr2
Sb02g031190.1
LOC_Os09g36730
65.4-
66.9 MB
20.4-
21.9 MB
Block score 978
b ZmMYB11
SbMYB23
OsMYB108
Sb Chr2
Zm Chr7
Sb02g031190
GRMZM2G000818
66.0-
66.5 MB
175.2-
176.5 MB
Block score 47
Os Chr9
Zm Chr7
LOC_Os09g36730
GRMZM2G000818
21.0-
21.4 MB
175.3-
175.5 MB
Block score 27
c ZmMYB42
SbMYB86
OsMYB102
Sb Chr7
Zm Chr4
Sb07g024890
GRMZM2G419239
59.6-
60.5 MB
215.0-
217.6 MB
Block score 35
Os Chr8
Zm Chr4
LOC_Os08g43550
GRMZM2G419239
27.4-
27.8 MB
214.8-
217.3 MB
Block score 21
Sb Chr7
Os Chr8
Sb07g024890
LOC_Os08g43550
59.5-
60.3 MB
27.3-
27.9 MB
Block score 421
d ZmMYB38
SbMYB86
OsMYB102
Sb Chr7
Zm Chr1
Sb07g024890
GRMZM2G084583
59.4-
60.5 MB
205.2
207.7 MB
Block score 868
Os Chr8
Zm Chr1
LOC_Os08g43550
GRMZM2G419239
27.4-
28.1 MB
203.6-
207.5 MB
Block score 218

SUPP. FIG. S1

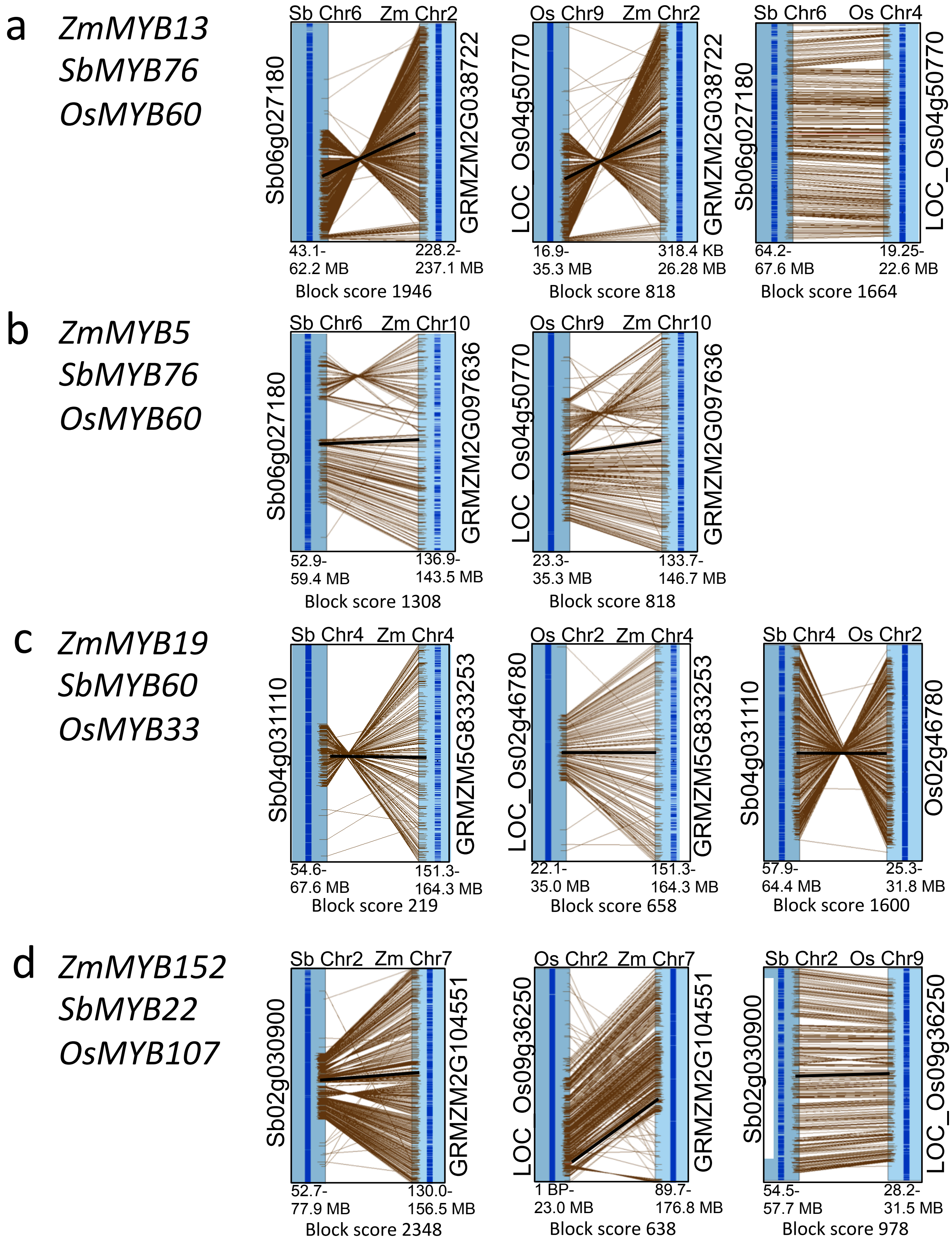
a
ZmMYB13
SbMYB76
OsMYB60
Sb Chr6 Zm Chr2
Sb06g027180
GRMZM2G038722
43.1-
62.2 MB
228.2-
237.1 MB
Block score 1946
Os Chr9 Zm Chr2
LOC_Os04g50770
GRMZM2G038722
16.9-
35.3 MB
318.4 KB
26.28 MB
Block score 818
Sb Chr6 Os Chr4
Sb06g027180
LOC_Os04g50770
64.2-
67.6 MB
19.25-
22.6 MB
Block score 1664
b
ZmMYB5
SbMYB76
OsMYB60
Sb Chr6 Zm Chr10
Sb06g027180
GRMZM2G097636
52.9-
59.4 MB
136.9-
143.5 MB
Block score 1308
Os Chr9 Zm Chr10
LOC_Os04g50770
GRMZM2G097636
23.3-
35.3 MB
133.7-
146.7 MB
Block score 818
c
ZmMYB19
SbMYB60
OsMYB33
Sb Chr4 Zm Chr4
Sb04g031110
GRMZM5G833253
54.6-
67.6 MB
151.3-
164.3 MB
Block score 219
Os Chr2 Zm Chr4
LOC_Os02g46780
GRMZM5G833253
22.1-
35.0 MB
151.3-
164.3 MB
Block score 658
Sb Chr4 Os Chr2
Sb04g031110
Os02g46780
57.9-
64.4 MB
25.3-
31.8 MB
Block score 1600
d
ZmMYB152
SbMYB22
OsMYB107
Sb Chr2 Zm Chr7
Sb02g030900
GRMZM2G104551
52.7-
77.9 MB
130.0-
156.5 MB
Block score 2348
Os Chr2 Zm Chr7
LOC_Os09g36250
GRMZM2G104551
1 BP-
23.0 MB
89.7-
176.8 MB
Block score 638
Sb Chr2 Os Chr9
Sb02g030900
LOC_Os09g36250
54.5-
57.7 MB
28.2-
31.5 MB
Block score 978

SUPP. FIG. S2

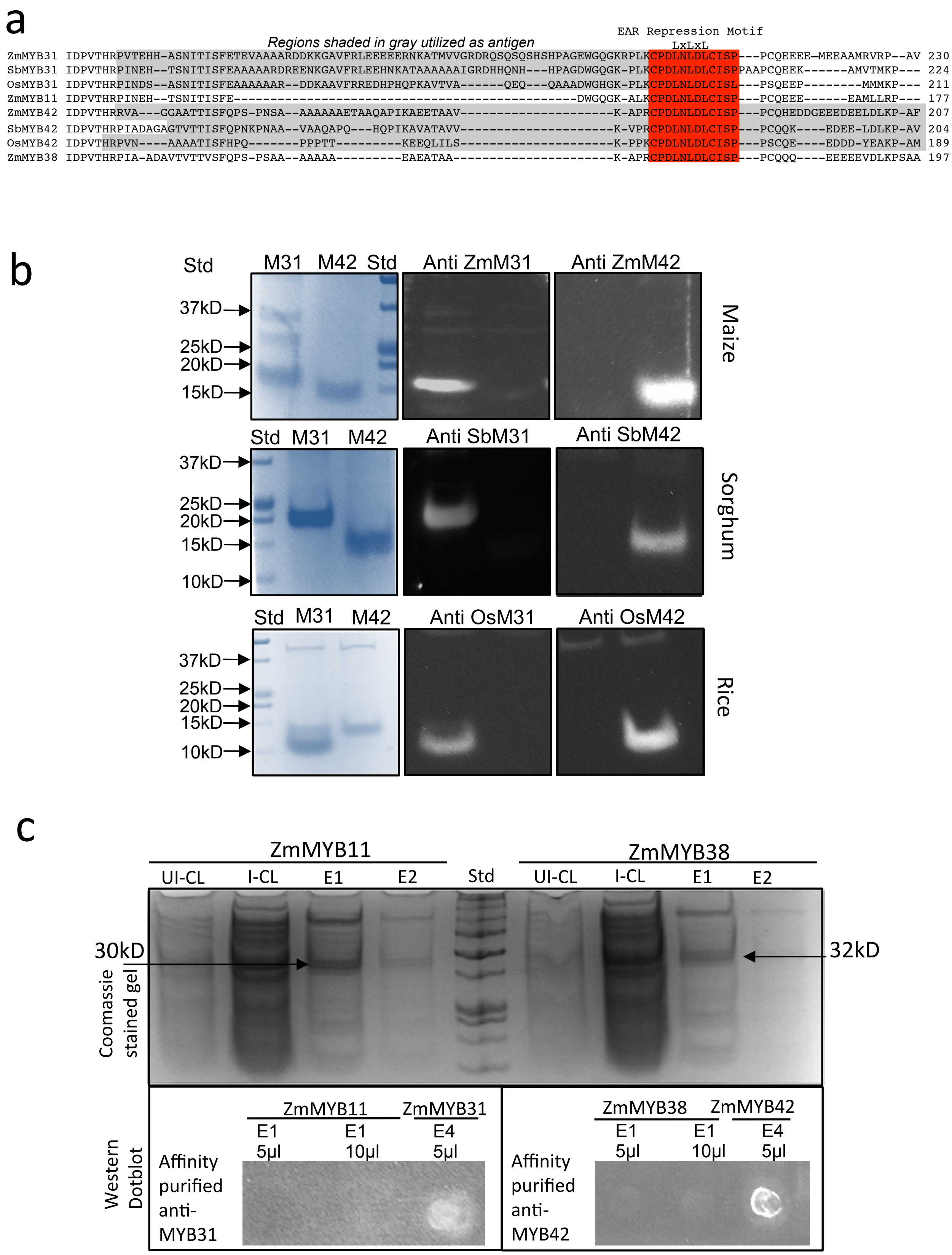

a
Regions shaded in gray utilized as antigen
EAR Repression Motif
LxLxL
ZmMYB31 IDPVTHRPVTEHH-ASNITISFETEVAAAARDDKKGAVFRLEEEEERNKATMVVGRDRQSQSQSHSHPAGEWGQGKRPLKCPDLNLDLCISP---PCQEEEE—MEEAAMRVRP--AV 230
SbMYB31 IDPVTHRPINEH--TSNITISFEAAAAARDREENKGAVFRLEEHNKATAAAAAAIGRDHHQNH---HPAGDWGQGK-PLKCPDLNLDLCISPPAAPCQEEK------AMVTMKP--- 224
OsMYB31 IDPVTHRPINDS--ASNITISFEAAAAAAR--DDKAAVFRREDHPHQPKAVTVA------QEQ---QAAADWGHGK-PLKCPDLNLDLCISL---PSQEEP--------MMMKP--- 211
ZmMYB11 IDPVTHRPINEH--TSNITISFE---DWGQGK-ALKCPDLNLDLCISP---PCQEEE------EAMLLRP--- 177
ZmMYB42 IDPVTHRRVA---GGAATTISFQPS-PNSA--AAAAAAETAAQAPIKAEETAAV---------------------K-APRCPDLNLDLCISP---PCQHEDDGEEEDEELDLKP-AF 207
SbMYB42 IDPVTHRPIADAGAGTVTTISFQPNKPNAA--VAAQAPQ---HQPIKAVATAVV---------------------K-VPRCPDLNLDLCISP---PCQQK----EDEE-LDLKP-AV 204
OsMYB42 IDPVTHRPVN----AAAATISFHPQ-------PPPTT---------KEEQLILS---------------------K-PPKCPDLNLDLCISP---PSCQE----EDDD-YEAKP-AM 189
ZmMYB38 IDPVTHRPIA-ADAVTVTTVSFQPS-PSAA--AAAAA---------EAEATAA----------------------K-APRCPDLNLDLCISP---PCQQQ----EEEEEVDLKPSAA 197
b
Std
M31
M42
Std
Anti ZmM31
Anti ZmM42
37kD
25kD
20kD
15kD
Maize
Std
M31
M42
Anti SbM31
Anti SbM42
37kD
25kD
20kD
15kD
10kD
Sorghum
Std
M31
M42
Anti OsM31
Anti OsM42
37kD
25kD
20kD
15kD
10kD
Rice
c
ZmMYB11
ZmMYB38
UI-CL
I-CL
E1
E2
Std
UI-CL
I-CL
E1
E2
30kD
32kD
Coomassie stained gel
Western Dotblot
ZmMYB11
ZmMYB31
E1 5µl
E1 10µl
E4 5µl
Affinity purified anti-MYB31
ZmMYB38
ZmMYB42
E1 5µl
E1 10µl
E4 5µl
Affinity purified anti-MYB42

SUPP. FIG. S3

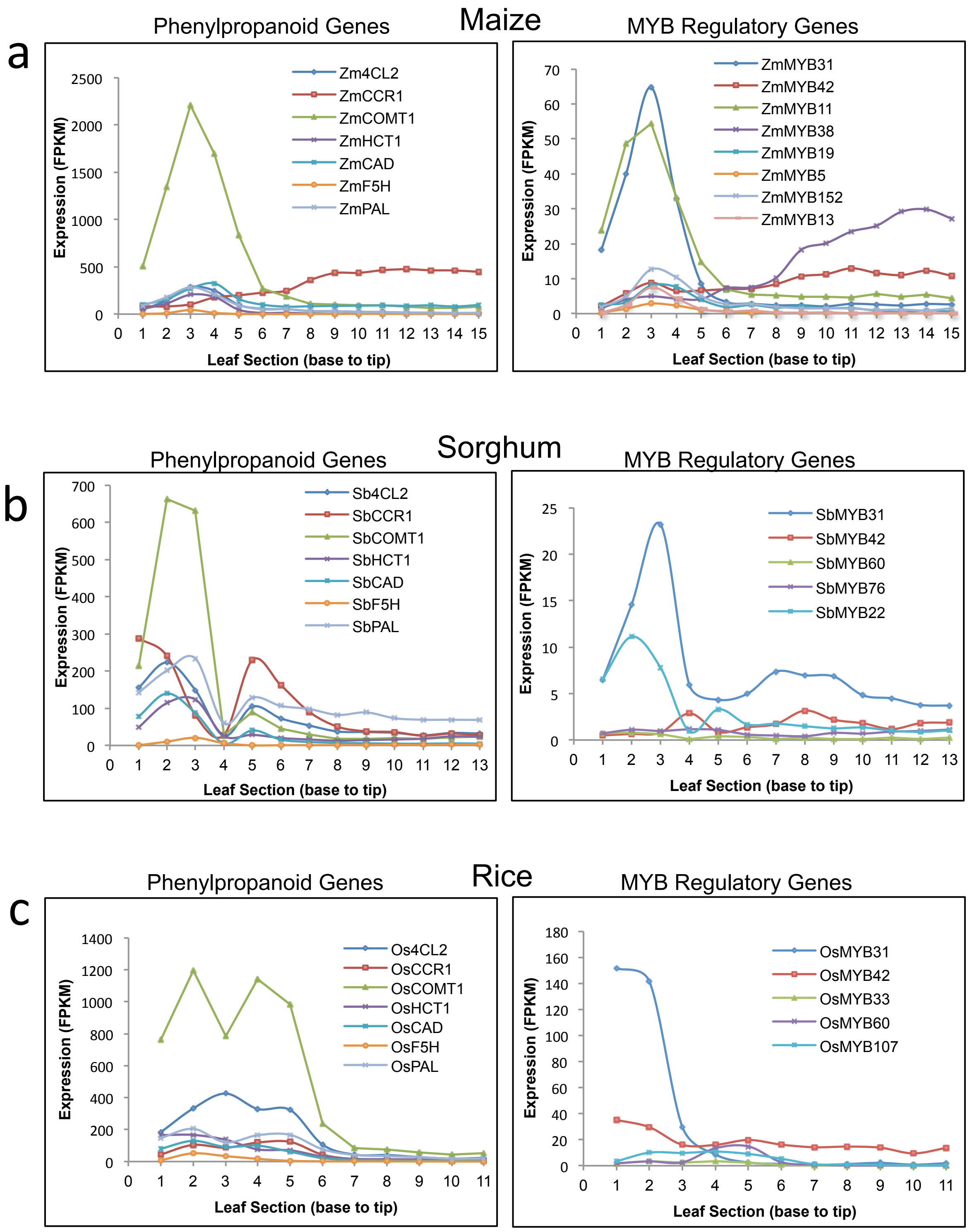

Maize
a
Phenylpropanoid Genes
Expression (FPKM)
Leaf Section (base to tip)
Zm4CL2
ZmCCR1
ZmCOMT1
ZmHCT1
ZmCAD
ZmF5H
ZmPAL
MYB Regulatory Genes
ZmMYB31
ZmMYB42
ZmMYB11
ZmMYB38
ZmMYB19
ZmMYB5
ZmMYB152
ZmMYB13
Sorghum
b
Phenylpropanoid Genes
Sb4CL2
SbCCR1
SbCOMT1
SbHCT1
SbCAD
SbF5H
SbPAL
MYB Regulatory Genes
SbMYB31
SbMYB42
SbMYB60
SbMYB76
SbMYB22
Rice
c
Phenylpropanoid Genes
Os4CL2
OsCCR1
OsCOMT1
OsHCT1
OsCAD
OsF5H
OsPAL
MYB Regulatory Genes
OsMYB31
OsMYB42
OsMYB33
OsMYB60
OsMYB107

SUPP. FIG. S4

Supplementary Table S1. List of gene-specific primers used for qRT-PCR expression analysis

Primer Name	Primer Sequence 5' – 3'	Gene Model #
ZmM31-R-RT-new	TAGAAGCGCGCTCATTTCAT	GRMZM2G050305
ZmM31-L-RT-new	GGACTGCAAGTGCAGCAG	
ZmM42-L-RTpcr	CAACGGGCACCACTTCCT	GRMZM2G419239
ZmM42-R-RTpcr	CCAACTAGCTCTATCCAAATCCA	
ZmCSE-L-RTpcr	ACGAGAACATCGAGCGTGTC	GRMZM2G418415
ZmCSE-R-RTpcr	TCCGAATGCTTATTCGACAG	
Zm4CL2-L3-RT	CACCGAATCCATCCCCAAGA	GRMZM2G075333
Zm4CL2-R3-RT	AGAAGAAGCTGCATACGGGC	
ZmCOMT1-L-RTpcr	GGTTCAAGGCCACCTACATC	AC196475.3_FG004
ZmCOMT1-R-RTpcr	GATACGAGGACCAAGCAAGC	
ZmF5H-L3-RT	CTTCGAGTTCCTGCCCTTCG	AC210173.4_FG005
ZmF5H-R3-RT	AGCGACCAGTTGAAGCCG	
ZmB-TUB-F-RT	CTACCTCACGGCATCTGCTATGT	GRMZM2G066191
ZmB-TUB-R-RT	GTCACACACACTCGACTTCACG	
SbM31-L4-RT	CAGCAACTTCCTCGGACTCA	Sb02g031190.1
SbM31-R4-RT	CGTATCATCCAGGCGAGATGT	
SbM42-L-RTpcr	CAGAGGCCTCGAGATGAAGT	Sb07g024890
SbM42-R-RTpcr	GAACCAAACCAAACGAAGGA	
SbCSE-L-RTpcr	CTGGCTCAACGAGAGATGC	Sb02g036570
SbCSE-R-RTpcr	GGAATCTGAACAGCCTGGAA	
Sb4CL2-L-RTpcr	GGTGTTCACTGAGGCCGTAT	Sb04g005210
Sb4CL2-R-RTpcr	CCTCTTCTGTTGCCCTTTCA	
SbCOMT1-L-RTpcr	GGTTCAAGGCCACCTACATC	Sb07g003860
SbCOMT1-R-RTpcr	GATGAGGACCATCCATGACA	
SbF5H-L3-RT	CAACTGCCCCCTGTACTGAA	Sb01g017270
SbF5H-R3-RT	AGCCCTCTAAACTCCCCACA	
SbUbiquitin-F	GCTGTACCTGCGTTTGTCTG	Sobic.004G050000
SbUbiquitin-R	ACACACGGGACACAAGACAC	
SbUBQ-F	CACTTCGACCGCCACTACT	Sb03g013260
SbUBQ-R	TTTCTCCTCTACGCCTGCTG	
OsM31-L-RT-new	CCAAGAGCACAGACTGCAAG	LOC_Os09g36730
OsM31-R-RT-new	AGGAGGAGGTGCTCATTTCA	
OsM42-L-RT-new	GCCGGCAACAACTTCCTC	LOC_Os08g43550
OsM42-R-RT-new	ACGATCATGGATTGGTTTGC	
OsCSE-L-RTpcr	GTGGCTCAACGAGAGATGC	LOC_Os07g37840
OsCSE-R-RTpcr	CTGAATAGAGTGGAGTGAATCCTG	
Os4CL2-L-RTpcr	CAAAAGCAGCTAAGGCCAAC	LOC_Os02g08100.1
Os4CL2-R-RTpcr	CTCCATTTTCATGGACAGCA	
OsCOMT1-L-RT-new	GCTTCAAGGCCACCTACATC	LOC_Os08g06100
OsCOMT1-R-RT-new	TTGATCGAGCTCAACAATGG	
OsF5H-RT-F5	GTGTGGTGTGTCATCCATGG	LOC_Os10g36848
OsF5H-RT-R5	CGCATGATTAGGACGGCC	
OsUBQ5-F-RT	ACCACTTCGACCGCCACTACT	LOC_Os01g22490
OsUBQ5-R-RT	ACGCCTAAGCCTGCTGGTT	

Supplementary Table S2. List of gene-specific primers used for ChIP-qRT-PCR binding analysis.

Primer name	Primer Sequence 5' - 3'	Gene Id
OsMYB31-F2	GGCAGTGTCAACACCATCAT	LOC_Os09g36730
OsMYB31-R2	GTGGAGCCTCTGCCTGATAA	
OsMYB42-F1	ATCCACCTCTCTTCCCATCC	LOC_Os08g4355
OsMYB42-R1	GGTTGGTTGGTGTTGGTCTC	
OsCSE_Cpcr_up2	CCGTGTAACAGTGCTCTCTTTTC	LOC_Os07g37840
OsCSE_Cpcr_dn2	GTTGGGTGGAACTGCTCGT	
Os4CL2-F2	AAAAGTCAGTGGCCTGAGAGA	LOC_Os02g08100.1
Os4CL2-R2	TGGTGACCATTGGCTTTTCT	
OsCOMT1-F2	CCAACTCCACACACTCCAAA	LOC_Os08g06100
OsCOMT1-R2	TTAAGTCGTGCATGGGGTTT	
OsF5H-F1	CCGGCTAATTGCCAGTTT	LOC_Os10g36848
OsF5H-R1	GGTTTGCCAATAGCATGGAA	
OsActin-F1	GTGTCCTGGTGATCCCCTTT	LOC_Os05g36290
OsActin-R1	CACAGTACTTACTGGCACGAACA	
SbMYB31-F1	CCAACTCACAGAGCCTTTGC	Sb02g031190.1
SbMYB31-R1	AAGCTAGGGTGGGTGAAGGT	
SbMYB42-F1	CAACTCACACCCACACAACC	Sb07g024890
SbMYB42-R1	GGCGAGAGATGAGAAGGAGA	
SbCSE_Cpcr_up2	TTCCGCTCCCTAATTCTGTG	Sb02g036570
SbCSE_Cpcr_dn2	GGTTTAGCCAGCTTGGAGGT	
Sb4CL2-F1	GAAGGTCAGGATGAACAATGG	Sb04g005210
Sb4CL2-R1	GAGCTGGAGGCTGAAGAGG	
SbCOMT1-F2	CGAGCACATCTCCCAAATCT	Sb07g003860
SbCOMT1-R2	TTTTCTGGTGGCGTTGTTG	
SbF5H-F1	GCGGACCATAACCCAGTTCT	Sb01g017270
SbF5H-R1	TTGTGGCTAGTGGTGATAGGAA	
SbActin-F1	GTTACTCCGCGCCAAACA	Sb09g021660
SbActin-R1	CCCAGGCAACTAGAGACCAA	
ZmM31-Cpcr-F2	CGCGATTCCTCAGTCAAGTG	GRMZM2G050305
ZmM31-Cpcr-R2	CTGCTGTGAGGTGCGAGC	
ZmM42-F1Cpcr	AATCCCCGCAAACTGTTCGT	GRMZM2G419239
ZmM42-R1Cpcr	GGTGGACGTGGACTGGAAC	
ZmCSE_Cpcr_up1	TGGAGTACCGTGCAACAGTG	GRMZM2G418415
ZmCSE_Cpcr_dn1	TTCCTTCATAGTTGGCACACG	
Zm4CL2-qPCR-up1	ACCACACAGCCGCTGATATT	GRMZM2G075333
Zm4CL2-qPCR-dn1	GGTGAGAGAGGGAGGGAGAG	
ZmCOMT1-qPCR-up1	GTGCGCGCATAGTAGTGAAA	AC196475.3_FG004
ZmCOMT1-qPCR-dn1	GTGCGAGGGGTTTTTATTGG	
ZmF5H-2F/2R	ATCCCCTTTTCATTTTCAGGCTCT	AC210173.4_FG005
ZmF5H-2F/2R	CTCCCTCCCCATCTCTCTATCTC	
ZmActin-F	TTTAAGGCTGCTGTACTGCTGTAGA	GRMZM2G126010
ZmActin-R	CACTTTCTGCTCATGGTTTAAGG	
ZmCopia-F	CGATGTGAAGACAGCATTCCT	AF398212.1
ZmCopia-R	CTCAAGTGACATCCCATGTGT	
-3-tubulinFW	AGGAGCCTCACGCAGATAAA	AC195340.3_FG001
-3-tubulinRV	CCTGCGGTAGAGGATGTTGT	

Supplementary Table S3. List of gene-specific primers used for cloning constructs

Primer Name	Primer Sequence 5' - 3'	Gene model
SbMYB31 Uni up	caccCCGGTGACACACCGCCCCATC	Sb02g031190.1
SbMYB31 Uni dn	tcaCTTGAGCGGCTTCCCCTGGCC	
SbMYB42 Uni up	caccGGCACCGTCACCACCATCTCG	Sb07g024890
SbMYB42 Uni dn	tcaGACGACGGCGGGCTTGAG	
OsMYB31 Uni up	caccGCCGATCAACGACAGCGCGTCC	LOC_Os09g36730
OsMYB31 Uni dn	tcaCTTGAGTGGCTTCCCATGGCCCC	
OsMYB42 Uni up	caccCACCGCCCCGTCAACGCCGCC	LOC_Os08g4355
OsMYB42 Uni dn	tcaCATCGCCGGCTTCGCCTCATAG	
ZmCSE_promF1	caccTTGTTGAACCGAATTTATAGAGAA	GRMZM2G418415
ZmCSE_promR1	CGTTTCGATGCGGGAGGG	
Zm4CL2promF1	caccAAAGACAAAGCTTCGGGGTG	GRMZM2G075333
Zm4CL2promR1	GGGCAGCTGGTTGGATGGT	
ZmF5HpromF1	AAACAAGATGCATAGTTGGC	AC210173.4_FG005
ZmF5HpromR1	ATTTCGTTTGGGTTTTAGTT	
ZmCOMT1promF1	CGGAAGAGACGGGTGGGTG	AC196475.3_FG004
ZmCOMT1promR1	ATGCGACGAAGAGGGACGAG	

Supplementary Table S4. List of maize, sorghum and rice syntelogs in the phenylpropanoid pathway and synteny scores. Synteny block scores were determined using the SyMAP v3.4 program[51] (ND = synteny not detected)

Gene	chr #	Maize gene Id	chr #	Gene	Sorghum gene Id	chr #	Gene	Rice gene Id	Synteny Block Score		
									maize-to-sorghum	maize-to-rice	sorghum-to-rice
Zm4CL1	1	GRMZM2G048522	7	*Sb4CL1*	Sb07g022040	6	*Os4CL1*	LOC_Os08g34790	868	115	298
Zm4CL2	5	GRMZM2G075333	4	*Sb4CL2*	Sb04g005210	2	*Os4CL2*	LOC_Os02g08100	554	316	836
ZmCCR1	1	GRMZM2G131205	7	*SbCCR1*	Sb07g021680	8	*OsCCR1*	LOC_Os08g34280	868	115	298
ZmCCR2	7	GRMZM2G131836	2	*SbCCR2*	Sb02g014910	2	*OsCCR2*	LOC_Os02g08420	819	ND	ND
ZmCOMT1	4	AC196475.3_FG004	7	*SbCOMT1*	Sb07g003860	8	*OsCOMT1*	LOC_Os08g06100	291	207	471
ZmCOMT2	4	GRMZM2G082007	4	*SbCOMT2*	Sb04g037820	2	*OsCOMT2*	LOC_Os02g57760	331	658	1600
ZmHCT1	5	GRMZM2G035584	4	*SbHCT1*	Sb04g025760	2	*OsHCT1*	LOC_Os02g39850	1938	1162	1600
ZmHCT2	2	GRMZM2G158083	6	*SbHCT2*	Sb06g021640	4	*OsHCT2*	LOC_Os04g42250	1946	1110	1664
ZmCAD	5	GRMZM5G844562	4	*SbCAD*	Sb04g005950	2	*OsCAD*	LOC_Os02g09490	554	316	836
ZmF5H	1	AC210173.4_FG005	1	*SbF5H*	Sb01g017270	10	*OsF5H*	LOC_Os10g36848	530	289	446
ZmPAL	4	GRMZM2G081582	4	*SbPAL*	Sb04g026520	2	*OsPAL*	LOC_Os02g41650	358	658	1600
ZmCSE	2	GRMZM2G418415	2	*SbCSE*	Sb02g036560	7	*OsCSE*	LOC_Os07g37840	1328	427	1006
ZmC3H	3	GRMZM2G138074	3	*SbC3H*	Sb03g037380	5	*OsC3H*	LOC_Os05g41440	2433	280	396
ZmMYB31	2	GRMZM2G050305	2	*SbMYB23*	Sb02g031190	9	*OsMYB108*	LOC_Os09g36730	1328	446	978
ZmMYB11	7	GRMZM2G000818	2	*SbMYB23*	Sb02g031190	9	*OsMYB108*	LOC_Os09g36730	47	27	ND
ZmMYB42	4	GRMZM2G419239	7	*SbMYB86*	Sb07g024890	8	*OsMYB102*	LOC_Os08g43550	35	21	421
ZmMYB38	1	GRMZM2G084583	7	*SbMYB86*	Sb07g024890	8	*OsMYB102*	LOC_Os08g43550	868	218	421
ZmMYB13	2	GRMZM2G038722	6	*SbMYB76*	Sb06g027180	4	*OsMYB60*	LOC_Os04g50770	1946	1110	1664
ZmMYB5	10	GRMZM2G097636	6	*SbMYB76*	Sb06g027180	4	*OsMYB60*	LOC_Os04g50770	1308	818	1664
ZmMYB19	4	GRMZM5G833253	4	*SbMYB60*	Sb04g031110	2	*OsMYB33*	LOC_Os02g46780	219	658	1600
ZmMYB152	7	GRMZM2G104551	2	*SbMYB22*	Sb02g030900	9	*OsMYB107*	LOC_Os09g36250	2348	638	978

Supplementary Table S5. Gene Coexpression Analysis in maize leaves. Summary of Spearman Correlation Coefficients (rho values) and Probability Values (p) reported in Fig. 3a.

			Spearman Correlation Coefficient (rho values)																				
Gene	Gene Model		4CL1	4CL2	CCR1	CCR2	F5H	COMT2	HCT1	HCT2	CAD	COMT1	PAL	CSE	C3H	MYB31	MYB42	MYB11	MYB38	MYB19	MYB5	MYB152	MYB13
4CL1	GRMZM2G048522			0.00	0.00	0.00	0.00	0.00	0.00	0.00	0.00	0.00	0.00	0.00	0.00	0.00	0.00	0.00	0.00	0.00	0.00	0.00	0.00
4CL2	GRMZM2G075333		NA		-0.86	0.90	0.98	-0.94	0.93	0.21	0.64	0.01	0.99	-0.12	0.65	0.76	-0.77	0.81	-0.89	0.94	0.96	0.93	0.98
CCR1	GRMZM2G131205		NA	0.00		-0.83	-0.90	0.89	-0.91	-0.07	-0.60	0.04	-0.89	-0.09	-0.74	-0.77	0.91	-0.79	0.93	-0.87	-0.81	-0.85	-0.85
CCR2	GRMZM2G131836		NA	0.00	0.00		0.94	-0.81	0.93	0.08	0.64	0.08	0.91	-0.05	0.69	0.93	-0.73	0.84	-0.86	0.94	0.80	0.85	0.89
COMT1	AC196475.3_FG004		NA	0.00	0.00	0.00		-0.90	0.96	0.15	0.69	0.03	0.98	-0.07	0.69	0.82	-0.80	0.79	-0.93	0.95	0.91	0.94	0.95
COMT2	GRMZM2G082007		NA	0.00	0.00	0.00	0.00		-0.88	-0.12	-0.65	-0.04	-0.93	-0.05	-0.62	-0.73	0.83	-0.88	0.88	-0.84	-0.94	-0.88	-0.93
HCT1	GRMZM2G035584		NA	0.00	0.00	0.00	0.00	0.00		0.19	0.60	0.04	0.95	0.02	0.67	0.84	-0.76	0.80	-0.90	0.95	0.89	0.89	0.93
HCT2	GRMZM2G158083	P values	NA	0.44	0.81	0.78	0.60	0.67	0.50		-0.18	0.38	0.21	0.13	-0.06	0.01	0.06	0.02	0.06	0.13	0.18	0.24	0.23
CAD	GRMZM5G844562		NA	0.01	0.02	0.01	0.00	0.01	0.02	0.52		0.34	0.64	0.08	0.55	0.68	-0.54	0.59	-0.60	0.57	0.66	0.65	0.56
F5H	AC210173.4_FG005		NA	0.98	0.90	0.78	0.92	0.88	0.88	0.16	0.22		0.01	0.45	-0.17	0.20	0.22	0.14	0.21	0.02	0.05	0.07	0.04
PAL	GRMZM2G081582		NA	0.00	0.00	0.00	0.00	0.00	0.00	0.46	0.01	0.96		-0.02	0.68	0.77	-0.80	0.82	-0.93	0.96	0.94	0.92	0.96
CSE	GRMZM2G418415		NA	0.67	0.74	0.86	0.81	0.86	0.94	0.65	0.77	0.09	0.94		0.01	0.20	0.03	0.25	0.00	-0.07	-0.09	-0.17	-0.13
C3H	GRMZM2G138074		NA	0.01	0.00	0.00	0.00	0.01	0.01	0.83	0.03	0.55	0.01	0.96		0.72	-0.75	0.61	-0.70	0.70	0.54	0.72	0.60
MYB31	GRMZM2G050305		NA	0.00	0.00	0.00	0.00	0.00	0.00	0.97	0.01	0.47	0.00	0.47	0.00		-0.65	0.83	-0.75	0.80	0.66	0.75	0.75
MYB42	GRMZM2G419239		NA	0.00	0.00	0.00	0.00	0.00	0.00	0.82	0.04	0.42	0.00	0.93	0.00	0.01		-0.70	0.90	-0.73	-0.69	-0.78	-0.74
MYB11	GRMZM2G000818		NA	0.00	0.00	0.00	0.00	0.00	0.00	0.95	0.02	0.63	0.00	0.38	0.02	0.00	0.00		-0.77	0.80	0.77	0.69	0.81
MYB38	GRMZM2G084583		NA	0.00	0.00	0.00	0.00	0.00	0.00	0.83	0.02	0.45	0.00	1.00	0.00	0.00	0.00	0.00		-0.89	-0.83	-0.82	-0.86
MYB19	GRMZM5G833253		NA	0.00	0.00	0.00	0.00	0.00	0.00	0.64	0.03	0.94	0.00	0.80	0.00	0.00	0.00	0.00	0.00		0.86	0.88	0.93
MYB5	GRMZM2G097636		NA	0.00	0.00	0.00	0.00	0.00	0.00	0.52	0.01	0.85	0.00	0.75	0.04	0.01	0.00	0.00	0.00	0.00		0.88	0.94
MYB152	GRMZM2G104551		NA	0.00	0.00	0.00	0.00	0.00	0.00	0.40	0.01	0.81	0.00	0.53	0.00	0.00	0.00	0.00	0.00	0.00	0.00		0.94
MYB13	GRMZM2G038722		NA	0.00	0.00	0.00	0.00	0.00	0.00	0.41	0.03	0.89	0.00	0.64	0.02	0.00	0.00	0.00	0.00	0.00	0.00	0.00	

Supplementary Table S6. Gene Coexpression Analysis in sorghum leaves. Summary of Spearman Correlation Coefficients (rho values) and Probability Values (p) reported in Fig. 3b.

			Spearman Correlation Coefficient (rho values)																	
**Gene**	**Gene Model**	**P values**	**4CL1**	**4CL2**	**CCR1**	**CCR2**	**COMT1**	**COMT2**	**HCT1**	**HCT2**	**CAD**	**F5H**	**PAL**	**CSE**	**C3H**	**MYB31**	**MYB42**	**MYB60**	**MYB76**	**MYB22**
4CL1	**Sb07g022040**			0.30	0.25	0.17	0.57	-0.65	0.67	0.59	0.47	0.41	0.27	0.30	0.11	0.30	-0.26	0.34	0.41	0.20
4CL2	**Sb04g005210**		0.32		0.95	0.92	0.77	-0.62	0.46	0.60	0.94	-0.23	0.96	-0.54	0.93	0.58	-0.72	0.68	-0.23	0.95
CCR1	**Sb07g021680**		0.42	0.00		0.85	0.75	-0.58	0.37	0.57	0.90	-0.43	0.87	-0.70	0.97	0.50	-0.63	0.58	-0.27	0.87
CCR2	**Sb02g014910**		0.58	0.00	0.00		0.71	-0.63	0.45	0.62	0.89	-0.15	0.95	-0.52	0.90	0.68	-0.71	0.74	-0.19	0.95
COMT1	**Sb07g003860**		0.04	0.00	0.00	0.01		-0.91	0.81	0.87	0.85	0.08	0.72	-0.34	0.67	0.40	-0.70	0.73	0.25	0.78
COMT2	**Sb04g037820**		0.02	0.03	0.04	0.02	0.00		-0.91	-0.95	-0.77	-0.21	-0.60	0.16	-0.53	-0.32	0.68	-0.79	-0.42	-0.59
HCT1	**Sb04g025760**		0.01	0.12	0.21	0.13	0.00	0.00		0.91	0.57	0.37	0.45	-0.04	0.32	0.09	-0.68	0.74	0.63	0.45
HCT2	**Sb06g021640**		0.03	0.03	0.04	0.03	0.00	0.00	0.00		0.72	0.15	0.58	-0.27	0.53	0.18	-0.73	0.88	0.49	0.57
CAD	**Sb04g005950**		0.10	0.00	0.00	0.00	0.00	0.00	0.04	0.01		-0.10	0.90	-0.38	0.85	0.63	-0.62	0.73	-0.05	0.88
F5H	**Sb01g017270**		0.17	0.45	0.14	0.62	0.80	0.49	0.21	0.62	0.73		-0.19	0.71	-0.47	0.20	0.08	0.09	0.56	-0.14
PAL	**Sb04g026520**		0.37	0.00	0.00	0.00	0.01	0.03	0.12	0.04	0.00	0.53		-0.47	0.90	0.63	-0.76	0.64	-0.24	0.95
CSE	**Sb02g036570**		0.32	0.06	0.01	0.07	0.25	0.59	0.89	0.36	0.20	0.01	0.11		-0.77	-0.05	0.53	-0.31	0.33	-0.52
C3H	**Sb03g037380**		0.72	0.00	0.00	0.00	0.01	0.06	0.28	0.06	0.00	0.11	0.00	0.00		0.51	-0.72	0.59	-0.34	0.88
MYB31	**Sb02g031190**		0.32	0.04	0.08	0.01	0.18	0.28	0.76	0.55	0.02	0.52	0.02	0.87	0.07		-0.22	0.23	-0.32	0.64
MYB42	**Sb07g024890**		0.38	0.01	0.02	0.01	0.01	0.01	0.01	0.01	0.02	0.79	0.00	0.06	0.01	0.47		-0.66	-0.06	-0.72
MYB60	**Sb04g031110**		0.26	0.01	0.04	0.00	0.00	0.00	0.00	0.00	0.01	0.76	0.02	0.31	0.03	0.46	0.01		0.29	0.66
MYB76	**Sb06g027180**		0.16	0.46	0.37	0.53	0.40	0.15	0.02	0.09	0.87	0.05	0.44	0.27	0.25	0.29	0.84	0.33		-0.20
MYB22	**Sb02g030900**		0.52	0.00	0.00	0.00	0.00	0.03	0.13	0.04	0.00	0.64	0.00	0.07	0.00	0.02	0.01	0.01	0.52	

Supplementary Table S7. Gene Coexpression Analysis in rice leaves. Summary of Spearman Correlation Coefficients (rho values) and Probability Values (p) reported in Fig. 3c.

			Spearman Correlation Coefficient (rho values)																	
**Gene**	**Gene Model**		**4CL1**	**4CL2**	**CCR1**	**CCR2**	**COMT1**	**COMT2**	**HCT1**	**HCT2**	**CAD**	**F5H**	**PAL**	**CSE**	**C3H**	**MYB31**	**MYB42**	**MYB33**	**MYB60**	**MYB108**
4CL1	**LOC_Os02g08100**			0.78	0.82	0.22	0.84	0.11	0.75	0.79	0.80	0.76	0.84	0.50	0.72	0.47	0.83	0.80	0.90	0.85
4CL2	**LOC_Os02g08100**		0.00		0.91	0.61	0.95	0.21	0.93	0.96	0.95	0.97	0.90	0.32	0.93	0.72	0.76	0.93	0.85	0.95
CCR1	**LOC_Os08g34280**		0.00	0.00		0.54	0.96	0.23	0.85	0.93	0.89	0.87	0.96	0.57	0.81	0.65	0.82	0.95	0.97	0.94
CCR2	**LOC_Os02g08420**		0.52	0.05	0.09		0.55	0.68	0.67	0.62	0.61	0.61	0.57	0.31	0.71	0.91	0.53	0.56	0.36	0.46
COMT1	**LOC_Os08g06100**		0.00	0.00	0.00	0.08		0.17	0.92	0.91	0.96	0.95	0.98	0.52	0.85	0.70	0.84	0.99	0.94	0.97
COMT2	**LOC_Os02g57760**		0.75	0.54	0.50	0.02	0.61		0.29	0.35	0.26	0.18	0.24	0.37	0.40	0.50	0.30	0.15	0.13	0.09
HCT1	**LOC_Os02g39850**		0.01	0.00	0.00	0.02	0.00	0.39		0.87	0.95	0.96	0.92	0.37	0.95	0.81	0.90	0.91	0.78	0.87
HCT2	**LOC_Os04g42250**	**P values**	0.00	0.00	0.00	0.04	0.00	0.28	0.00		0.90	0.90	0.89	0.39	0.91	0.67	0.76	0.88	0.87	0.90
CAD	**LOC_Os02g09490**		0.00	0.00	0.00	0.05	0.00	0.43	0.00	0.00		0.96	0.93	0.48	0.92	0.75	0.82	0.94	0.85	0.95
F5H	**LOC_Os10g36848**		0.01	0.00	0.00	0.05	0.00	0.59	0.00	0.00	0.00		0.91	0.27	0.92	0.74	0.81	0.94	0.82	0.94
PAL	**LOC_Os02g41650**		0.00	0.00	0.00	0.07	0.00	0.48	0.00	0.00	0.00	0.00		0.56	0.83	0.71	0.90	0.97	0.94	0.92
CSE	**LOC_Os07g37840**		0.12	0.34	0.07	0.36	0.10	0.26	0.26	0.23	0.13	0.42	0.07		0.35	0.42	0.53	0.46	0.59	0.46
C3H	**LOC_Os05g41440**		0.01	0.00	0.00	0.01	0.00	0.22	0.00	0.00	0.00	0.00	0.00	0.30		0.80	0.83	0.81	0.73	0.84
MYB31	**LOC_Os09g36730**		0.14	0.01	0.03	0.00	0.02	0.12	0.00	0.02	0.01	0.01	0.01	0.20	0.00		0.72	0.72	0.54	0.64
MYB42	**LOC_Os08g43550**		0.00	0.01	0.00	0.10	0.00	0.37	0.00	0.01	0.00	0.00	0.00	0.10	0.00	0.01		0.82	0.81	0.75
MYB33	**LOC_Os02g46780**		0.00	0.00	0.00	0.07	0.00	0.66	0.00	0.00	0.00	0.00	0.00	0.15	0.00	0.01	0.00		0.92	0.96
MYB60	**LOC_Os04g50770**		0.00	0.00	0.00	0.27	0.00	0.71	0.00	0.00	0.00	0.00	0.00	0.06	0.01	0.09	0.00	0.00		0.93
MYB107	**LOC_Os09g36250**		0.00	0.00	0.00	0.15	0.00	0.79	0.00	0.00	0.00	0.00	0.00	0.15	0.00	0.04	0.01	0.00	0.00	

Supplementary Table S8: Correlation between MYB31 and MYB42 mRNA accumulation and phenypropanoid target gene mRNA accumulation in leaves of maize, sorghum and rice. Pearson Product-Moment Correlation Coefficients (r) were calculated using the qRT-PCR generated for Fig. 4b-g. Three biological replicates were performed with three technical replicates each. The two-tailed probability (p) was calculated from the Pearson correlation coefficient (n = 3).

	**MYB31**					
	**Maize**		**Sorghum**		**Rice**	
Gene	r	p	r	p	r	p
COMT	-0.48	0.68	1.00	0.05	1.00	0.02
CSE	0.80	0.42	-0.62	0.57	-0.36	0.77
4CL2	-0.55	0.63	-0.76	0.45	0.99	0.11
F5H	0.87	0.33	-0.42	0.72	0.90	0.28
	**MYB42**					
	**Maize**		**Sorghum**		**Rice**	
	r	p	r	p	r	p
COMT	-0.98	0.13	-0.91	0.27	0.99	0.11
CSE	0.05	0.97	0.17	0.27	-0.53	0.64
4CL2	-0.99	0.08	0.35	0.77	0.99	0.23
F5H	0.94	0.22	-0.07	0.96	0.80	0.41

Supplementary Table S9: Summary of MYB31 and MYB43 TF binding to target phenylpropanoid and regulatory genes in maize, sorghum and rice. The results of ChIP-qPCR assays in which significant enrichment was detected in at least two of three biological replicates are shown (in bold letters). Three technical replicates (n = 3) were performed for each biological replicate (R). Values shown are average enrichment for control and test genes relative to the idiotypic antibody control, std deviation, and P values. The P values are highlighted in bold if less than 0.1. FC p indicates the p value derived using Fisher's combined probability test.

Basal Leaf Tissue

MYB31

Target Gene	R1 Avg	R1 Stdev	R1 p	R2 Avg	R2 Stdev	R2 p	R3 Avg	R3 Stdev	R3 p	FC p
OsAct	0.230	0.040		3.100	0.370		1.460	0.950		
OsCse	1.950	1.910	0.130	6.690	2.900	**0.090**	3.420	0.640	**0.047**	**0.027**

MYB42

Target Gene	R1 Avg	R1 Stdev	R1 p	R2 Avg	R2 Stdev	R2 p	R3 Avg	R3 Stdev	R3 p	FC p
ZmTub	0.710	0.500		0.900	0.700		1.200	0.150		
Zm4Cl2	9.840	4.600	**0.030**	8.130	1.900	**0.004**	2.510	1.200	**0.080**	**0.001**
ZmTub	0.710	0.500		0.900	0.700					
ZmMyb42	4.950	0.000	**0.002**	8.200	0.500	**0.001**	8.930	9.650	0.150	**0.000**
OsAct	0.890	0.120		0.630	0.260		2.570	0.790		
OsMyb42	0.490	0.180	0.060	14.380	10.050	**0.070**	14.060	7.200	**0.054**	**0.010**

Mid Leaf Tissue

MYB31

Target Gene	R1 Avg	R1 Stdev	R1 p	R2 Avg	R2 Stdev	R2 p	R3 Avg	R3 Stdev	R3 p	FC p
ZmCopia	1.020	0.060		2.540	0.520		1.430	0.270		
ZmCse	2.460	0.300	**0.004**	0.720	0.050	0.040	2.460	0.300	**0.001**	**0.000**
SbAct	0.865	0.170		0.200	0.030		1.800	0.260		
SbCse	3.810	1.040	**0.025**	0.480	0.070	0.020	8.700	2.700	**0.025**	**0.005**
OsAct	1.630	0.300		0.660	0.360		1.150	0.140		
OsCse	3.400	1.300	**0.070**	3.020	1.380	**0.050**	0.730	0.580		**0.023**
ZmAct	0.670	0.470		0.430	0.250		1.420	0.270		
Zm4Cl2	1.200	0.890	**0.080**	1.160	0.870	0.147	5.600	0.840	**0.006**	**0.004**
SbAct	0.870	0.170		0.190	0.030		1.770	0.260		
Sb4Cl2	18.100	6.410	**0.020**	0.810	0.190	**0.020**	1.170	0.190	**0.070**	**0.002**
OsAct	1.630	0.310		1.130	0.540		1.150	0.140		
OsF5H	6.560	4.670	**0.090**	9.730	3.000	**0.010**	1.490	0.150	**0.050**	**0.003**
OsAct	1.630	0.310		1.128	0.540		1.150	0.140		
OsMyb42	3.730	1.340	**0.042**	1.460	0.590	0.249	3.430	0.750	**0.018**	**0.006**

MYB42

Target Gene	R1 Avg	R1 Stdev	R1 p	R2 Avg	R2 Stdev	R2 p	R3 Avg	R3 Stdev	R3 p	FC p
SbAct	0.680	0.160		0.650	0.110		1.850	1.240		
SbCse	1.310	0.290	**0.053**	0.960	0.460	0.140	12.680	1.610	**0.010**	**0.005**
OsAct	1.380	0.110		3.480	2.110		1.090	0.140		
OsCse	2.100	0.150	**0.012**	16.400	3.270	**0.009**	1.060	0.380	0.475	**0.001**
SbAct	0.670	0.160		0.640	0.110		1.850	1.240		
Sb4Cl2	8.650	4.200	**0.040**	1.220	0.390	**0.089**	1.120	0.520	0.160	**0.024**
SbAct	0.680	0.160		0.650	0.110		1.860	1.240		
SbF5H	1.200	0.260	**0.070**	1.180	0.320	**0.080**	0.850	0.130	0.130	**0.035**
OsAct	1.380	0.110		0.470	0.230		1.100	0.140		
OsF5H	2.170	0.160	**0.020**	15.870	3.600	**0.010**	1.190	0.130	0.260	**0.002**
ZmAct	1.890	0.850		0.370	0.160		1.650	0.520		
ZmMyb42	1.010	0.230	0.120	1.210	0.430	**0.063**	3.530	0.810	**0.060**	**0.025**

Leaf Tip Tissue

MYB31

Target Gene	R1 Avg	R1 Stdev	R1 p	R2 Avg	R2 Stdev	R2 p	R3 Avg	R3 Stdev	R3 p	FC p
ZmCopia	1.630	0.300		1.880	1.430		0.190	0.070		
ZmComt1	14.220	1.100	**0.002**	1.950	0.780	0.450	2.180	0.710	**0.020**	**0.000**
SbAct	0.660	0.200		0.110	0.110		1.660	0.350		
SbComt1	2.670	1.060	**0.040**	1.000	0.260	**0.010**	0.980	0.780	**0.100**	**0.002**
ZmCopia	0.840	0.400		1.290	0.230		0.190	0.001		
ZmCse	1.900	0.290	**0.009**	4.790	1.880	**0.040**	0.320	0.180	0.170	**0.003**
OsAct	1.300	0.910		0.870	0.560		2.700	0.730		
OsCse	4.800	2.310	**0.046**	1.420	1.000	0.300	25.190	20.000	**0.090**	**0.027**
SbActin	1.780	0.250		2.300	0.480		1.070	0.350		
Sb4Cl2	74.680	47.890	**0.059**	11.170	5.780	**0.070**	2.780	1.110	**0.040**	**0.008**
OsAct	1.930	0.200		1.150	0.210		0.220	0.020		
OsMyb31	2.010	1.180	0.440	4.210	1.250	**0.030**	1.790	0.320	**0.010**	**0.003**
OsAct	1.900	0.200		1.160	0.210		0.220	0.020		
OsMyb42	1.580	2.260	0.410	3.840	1.110	**0.020**	1.560	0.390	**0.002**	**0.000**

MYB42

Target Gene	R1 Avg	R1 Stdev	R1 p	R2 Avg	R2 Stdev	R2 p	R3 Avg	R3 Stdev	R3 p	FC p
SbAct	1.510	0.310		0.530	0.190		1.830	0.260		
SbComt1	3.960	1.340	**0.045**	3.290	0.620	**0.010**	1.430	0.330	**0.110**	**0.003**
OsActin	0.930	0.030		2.210	0.260		0.840	0.170		
OsComt1	2.900	0.350	**0.005**	7.460	0.840	**0.003**	2.640	1.280	**0.050**	**0.000**
SbAct	1.500	0.310		0.530	0.190		1.830	0.260		
SbCse	3.740	0.300	**0.010**	4.360	2.830	**0.080**	2.970	0.160	**0.020**	**0.001**
OsAct	0.410	0.450		2.500	1.420		1.890	0.420		
OsCse	1.210	0.970	**0.065**	17.480	10.190	**0.050**	1.630	1.200	0.327	**0.022**
SbAct	0.710	0.100		0.920	0.450		0.550	0.120		
Sb4Cl2	68.230	29.090	**0.028**	1.890	0.970	0.150	2.030	0.710	**0.030**	**0.007**
OsAct	0.410	0.450		2.500	1.420		1.890	0.420		
Os4Cl2	2.030	0.330	**0.001**	3.280	5.500	0.400	27.980	2.120	**0.001**	**0.000**
ZmTub	0.840	0.410		1.290	0.230		0.190	0.001		
ZmMyb31	6.020	0.020	**0.001**	6.030	1.090	**0.007**	0.080	0.030	0.020	**0.000**
OsAct	0.930	0.030		2.210	0.260		0.840	0.170		
OsMyb31	2.620	0.880	**0.040**	3.380	0.910	**0.070**	3.940	1.070	**0.024**	**0.004**
SbAct	0.700	0.100		1.380	0.100		0.550	0.120		
SbMyb42	7.350	2.340	**0.020**	0.930	0.080	0.001	2.000	0.970	**0.050**	**0.000**
